# Seasonality Affects the Diversity and Composition of Bacterioplankton Communities in Dongjiang River, a Drinking Water Source of Hong Kong

**DOI:** 10.3389/fmicb.2017.01644

**Published:** 2017-08-31

**Authors:** Wei Sun, Chunyu Xia, Meiying Xu, Jun Guo, Guoping Sun

**Affiliations:** ^1^Guangdong Provincial Key Laboratory of Microbial Culture Collection and Application, Guangdong Institute of Microbiology Guangzhou, China; ^2^School of Life Sciences, Longyan University Longyan, China; ^3^State Key Laboratory of Applied Microbiology Southern China Guangzhou, China

**Keywords:** bacterioplankton, microbial community, seasonal effects, Dongjiang River, drinking water source

## Abstract

Water quality ranks the most vital criterion for rivers serving as drinking water sources, which periodically changes over seasons. Such fluctuation is believed associated with the state shifts of bacterial community within. To date, seasonality effects on bacterioplankton community patterns in large rivers serving as drinking water sources however, are still poorly understood. Here we investigated the intra-annual bacterial community structure in the Dongjiang River, a drinking water source of Hong Kong, using high-throughput pyrosequencing in concert with geochemical property measurements during dry, and wet seasons. Our results showed that *Proteobacteria, Actinobacteria*, and *Bacteroidetes* were the dominant phyla of bacterioplankton communities, which varied in composition, and distribution from dry to wet seasons, and exhibited profound seasonal changes. *Actinobacteria, Bacteroidetes*, and *Cyanobacteria* seemed to be more associated with seasonality that the relative abundances of *Actinobacteria*, and *Bacteroidetes* were significantly higher in the dry season than those in the wet season (*p* < 0.01), while the relative abundance of *Cyanobacteria* was about 10-fold higher in the wet season than in the dry season. Temperature and ${\text{NO}}_{3}^{-}$-N concentration represented key contributing factors to the observed seasonal variations. These findings help understand the roles of various bacterioplankton and their interactions with the biogeochemical processes in the river ecosystem.

## Introduction

Microorganisms, the most abundant and diverse organisms on Earth, contribute to many biogeochemical cycles, such as carbon, and nitrogen cycles (Falkowski et al., 2008). In aquatic systems, bacterioplankton play critical roles in the ecological function by conducting a broad array of chemical transformations (e.g., decomposition of organic matters, OM), which sustain a balance between the phytoplankton (i.e., OM consumption), and other primary producers that produce organic matters (Azam and Worden, 2004). Bacterioplankton are highly dynamic in composition and structure in response to different environmental gradients across ecosystems (Andersson et al., 2010; Comte and Del Giorgio, 2010; Ruiz-Gonzalez et al., 2013), which may result in diversified functional roles in the biogeochemical processes. Therefore, understanding how bacterioplankton communities respond to dynamic environmental properties could provide clues to underlie mechanisms that regulate community assembly (Green et al., 2008).

Serving as drinking water sources, large river ecosystems are indispensable for socioeconomic activities, household activities, and the development of civilizations (Houhou et al., 2016). Various temporal and spatial changes in large rivers create unique habitats where bacterioplankton develop complex groups, and these groups are directly involved in biogeochemical processes. Previous studies on bacterioplankton in rivers have been focusing on pathogens (Nawab et al., 2016). Although, a great attention has been paid to non-fecal opportunistic pathogens and biogeochemical process-related microorganisms in recent years (Proctor and Hammes, 2015), the understanding of variation patterns of large river microbial communities, and their responses to changes of natural environmental factors remain limited (Jackson et al., 2014). In recent years, the development of high-throughput molecular technologies has extended the bioinformatics data we can acquire by several orders of magnitude than the traditional approaches, with the possibility of obtaining more comprehensive, and detailed information about the characteristics of river microbial communities.

The variations of bacterioplankton resulting from temporal changes are more significant than those from spatial changes (Leff et al., 1999), especially in places where the climate changes drastically between seasons. It has been indicated that microbial communities follow annually reoccurring patterns and exhibit a foreseeable temporal pattern within a single habitat (Portillo et al., 2012). Although, recent advances in freshwater microbial communities have found that environment factors such as temperature, flow rate, dissolved organic matter (DOM), and nitrogen concentrations are related to the temporal shift of freshwater bacterioplankton (Yannarell et al., 2003; Zwisler et al., 2003; Crump and Hobbie, 2005; Crump et al., 2009; Adams et al., 2010; Portillo et al., 2012), few studies attempted to interpret the shifts of bacterioplankton communities in river ecosystems in combination of both environmental factors, and the seasonality. The Dongjiang River has an important economic and ecological status as a major water reservoir for the south China. Front- and typhoon-type rainfalls and large seasonal variations in rainfall and runoff dominate the basin around. We have previously analyzed bacterioplankton characteristics in the Dongjiang River using DGGE and 16S rRNA profiling (Liu et al., 2012). However, the composition, and structure of bacterioplankton communities and their dynamic changes over time are not included. It is known that the climate along the Dongjiang River where abundant planktonic microorganisms are present changes with seasons (Liu et al., 2010), and these changes are expected to severely impact processes of the nutrient turnover in the river. As a result, the bacterioplankton structure, and their ecological functions are modulated. Consequently, it is important to correlate variations of planktonic communities to seasonal changes and to understand how the bacterioplankton manage to adapt to them.

High-throughput sequencing is helpful to obtain detailed information about the temporal changes of bacterioplankton in river ecosystems. In the present study, we hypothesized that the bacterioplankton communities could sensitively respond to the physiochemical fluctuations along the Dongjiang River, and exhibit pronounced seasonal shifts in composition, and structure. We examined the temporal variability in bacterioplankton community structure and composition along the Dongjiang River using barcoded 454 pyrosequencing in concert with traditional microbiological analyses and geochemical measurements. Our results supported that hypothesis by revealing a profound seasonal pattern, and highlighted the roles of temperature and ${\text{NO}}_{3}^{-}$-N concentration in reshaping the bacterioplankton.

## Materials and methods

### Sample collection and physicochemical analysis

The Dongjiang River is located in area with large seasonal variations in rainfall and runoff. The river has a coverage of 35,340 km^2^, of which 90% belong to the Guangdong province (Liu et al., 2010). Four cities along the mainstream: Heiyuan (HY), Guozhu (GZ), Huizhou (HZ), and Qiaotou (QT) were chosen as sampling sites. Water samples were collected using plexiglass water sampler at 3–5 m in depth from the Dongjiang River in dry (March) and wet (September) seasons in 2011, stored in sterile plastic bags, and deposited in an ice box. All samples were transferred to the lab within 10 h and stored at 4°C before any further treatment. Subsamples of ~1–2 L for each water sample were achieved by filtering through 0.22 μm flat filters within 12 h after water collection and were kept at −80°C for genome extraction (Eiler et al., 2012; Portillo et al., 2012).

Water quality parameters such as pH, dissolved oxygen (DO), and temperature were monitored *in situ*. Ammonia-N (${\text{NH}}_{4}^{+}$-N), nitrite-N (${\text{NO}}_{2}^{-}$-N), and nitrate-N (${\text{NO}}_{3}^{-}$-N) were measured following the methods described by Sun et al. (2016). Permanganate index (PI) and total suspended solids (TSS) were quantified using the methods of ISO 11923-1997 and ISO 8467-1993. Total nitrogen (TN), total carbon (TC), total organic carbon (TOC), and total inorganic carbon (TIC) were estimated by the TOC Analyzer (Elementar, Germany). All measurements were conducted in triplicate.

### Genome extraction, 16s rDNA amplification and pyrosequencing

Genomic DNA was extracted from each water sample as described before (Sun et al., 2014), and the concentration and quality were determined using photometrical BioSpec-nano (Shimazu, Japan). The rest was stored at −30°C. The V1-V3 hypervariable region of 16S rRNA genes was amplified using the primer set 5F (TGG AGA GTT TGA TCC TGG CTC AG) and 534R (TAC CGC GGC TGC TGG CAC) with a 7-bp barcode representing each sample. Each PCR mixture (25 μl) contained 12.5 μl of Gotaq Hotstart polymerase 2 × mix (Promega, USA), 1 μl of each primer (5 μM), 1 μl genomic DNA (0.1–10 ng μl^−1^), and 9.5 μl distilled H_2_O. Reactions were cycled with an initial denaturation at 95°C for 2 min; 25 cycles of 94°C for 30 s, 56°C for 25 s, and 72°C for 25 s; with a final extension at 72°C for 5 min. The PCR products were purified by QIAquick Gel extraction kit (Qiagen, CA, USA). Pyrosequencing of the 16S rRNA gene amplicon was performed on a Genome Sequencer FLX Titanium (Roche, NJ, USA), and 400 bp-long reads on average were produced from the forward direction 5F. The 454 sequence reads have been archived in the NCBI Short Read Archive under accession number SRR5027088.

### Processing of pyrosequencing data

To improve reads quality and minimize the random sequencing errors (Kunin et al., 2010), the raw sequence data were firstly filtered using the Ribosomal Database Project website to remove sequences shorter than 150 bp, with an average Q-score lower than 25, with an ambiguous base-call (N), or without the primer sequence (Yang et al., 2012). The trimmed sequences were sorted by the barcode sequences, which were truncated together with the primers later on. The pre-clustering step in MOTHUR was performed to reduce sequencing noise (Schloss et al., 2009).

### Assignment of operational taxonomic units (OTUs) and microbial taxonomic classification

The assignation of operational taxonomic units (OTUs, 97% similarity), species diversity, and richness estimators (Shannon Index, Simpson Index, ACE and Chao1), taxonomic assignment, and comparisons of community structure were analyzed by MOTHUR (Schloss et al., 2009). The processing details were conducted as described previously (Yang et al., 2012). RDP Classifier (Ver. 2.1) was used as reference to phylogenetically assign the taxonomic classifications to each representative sequences (Cole et al., 2009). The comparisons in community structure within a single sample and those pairwise were determined by FastUniFrac metric (Hamady et al., 2010). The resulting Bray-Curtis distance metrics were applied to explicit the pairwise community structure among the samples according to the Principle Coordinates Analysis (PCoA) (Lozupone and Knight, 2005), and Non-metric multidimensional scaling (NMDS) analyses.

### Statistical analysis

Environmental variables were firstly normalized to a common scale (through the subtraction of the mean and division of the standard deviation), and environmental classification was then conducted by hierarchical clustering with the Minitab statistical software (release 16; Minitab, State College, PA, USA) using Euclidean distance between samples (Magalhães et al., 2008). In order to examine whether bacterioplankton communities were significantly different between wet and dry seasons, three different non-parametric analyses for multivariate data were conducted including analysis of similarities (ANOSIM) (Clarke, 1993), non-parametric multivariate analysis of variance (adonis) (Anderson, 2001), and multiresponse permutation procedure (MRPP) (Mielke et al., 1981). These analyses were conducted with the R v.2.8.1, vegan package (Dixon, 2003).

The response ratios (RR) were computed to show the difference of phylogenetic composition and structure of microbial communities between the dry and wet seasons (He et al., 2012). The RR of each variable was calculated by dividing the mean of the wet season communities to that of the dry season communities. The 95% confident interval for each response variable was determined, and the statistical difference of communities between the wet and dry seasons was estimated.

The correlations between the environmental factors and bacterioplankton communities of different taxonomic levels were performed by redundancy analysis (RDA). The relationships between the bacterioplankton communities and physiochemical properties of water column were evaluated to elucidate the inter-relationships among environmental factors and bacterioplankton communities. First, the most correlated parameters were selected by BioENV procedure (Clarke and Ainsworth, 1993), including three physical parameters TSS, pH, and temperature and three chemical parameters ${\text{NH}}_{4}^{+}$-N, ${\text{NO}}_{3}^{-}$-N, and TOC, respectively. Then, the partial Mantel test was conducted to control possible co-varying effects between physical, and chemical parameters. All above were performed with the R. Furthermore, the relationships between the taxonomic diversity for the group with physicochemical water features were tested with linear regression analyses using SPSS 17.0.

## Results

### Effects of seasonality on water physicochemical properties

**Figure 1:**
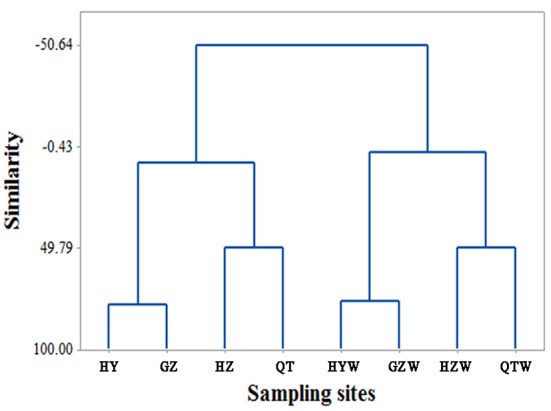
Hierarchical clustering dendrogram of environment factors constructed using Euclidean distance and Ward linkage of all water physicochemical factors in the Dongjiang River.

Multivariate clustering of river-water physicochemical factors (Table 1) identified two clusters of environmental conditions: one from dry season samples and the other from wet season samples (Figure 1), which were significantly different (*p* < 0.05), suggesting seasonal changes of water quality in the Dongjiang River. Moreover, the samples from upstream sites HY (HYW), and GZ (GZW) were separated from the downstream ones (HZ, HZW, QT, and QTW) in both seasons (Figure 1). Turbidity, TSS and the contents of different nitrogen, and carbon forms were lower in the upstream sites HY (HYW), and GZ (GZW) than in the downstream sites HZ (HZW), and QT (QTW) as shown in Table 1, therefore water qualities of the upstream sites were better than those of the downstream sites, which correlates well with the increasing population sizes, and economic development styles from the nearby city of Huizhou and Qiaotou.

**Table 1:** Physical and chemical properties of the water column in the Dongjiang River.

**Environmental factors^a^**	**Sampling sites**							
	**HY^b^**	**GZ^b^**	**HZ^b^**	**QT^b^**	**HYW^c^**	**GZW^c^**	**HZW^c^**	**QTW^c^**
Time	Mar.	Mar.	Mar.	Mar.	Sep.	Sep.	Sep.	Sep.
Longitude (°E)	114.701	114.689	114.408	114.104	114.701	114.689	114.408	114.104
Latitude (°N)	23.730	23.518	23.098	23.051	23.730	23.518	23.098	23.051
Distance (kilometer)^d^	-	28.24	93.98	42.29	-	28.24	93.98	42.29
**PHYSICAL**								
pH	6.50	6.67	6.58	7.20	7.31	7.39	6.88	7.02
DO (mg/L)	9.78	8.90	7.20	7.80	7.45	8.26	4.88	3.96
Turbidity (NTU)	3.20	8.60	18.50	25.80	4.13	14.93	16.33	27.63
Tem (°C)	16.1	15.9	17.3	17.5	23.5	21.6	25.7	26.4
TSS (mg/L)	15.0	25.0	28.3	75.0	14.5	20.0	24.4	26.7
**NITROGEN**								
${\text{NH}}_{4}^{+}$-N (mg/L)	0.18	0.27	2.19	1.13	0.25	0.19	1.04	0.31
${\text{NO}}_{2}^{-}$-N (mg/L)	0.011	0.029	0.065	0.080	0.008	0.033	0.188	0.053
${\text{NO}}_{3}^{-}$-N (mg/L)	0.92	1.32	1.42	1.38	0.76	1.12	1.72	2.64
DIN (mg/L)	1.117	1.622	3.675	2.588	1.020	1.338	2.960	3.001
TN (mg/L)	5.15	6.47	9.78	9.19	1.49	1.69	3.14	3.15
**CARBON**								
TIC (mg/L)	7.30	7.38	8.78	8.67	4.81	4.96	6.22	5.93
TOC (mg/L)	4.70	4.86	5.80	5.39	2.08	1.95	2.83	2.56
TC (mg/L)	12.00	12.24	14.58	14.06	6.89	6.91	9.05	8.49
PI (mg/L)	3.86	3.29	3.63	3.13	2.17	1.59	3.34	1.99

a *DO, dissolved oxygen; TSS, total suspended solids; TIC, total inorganic carbon; TOC, total organic carbon; TC, total carbon; TN, total nitrogen; PI, permanganate index*.

b *Water samples in March; XF, Xinfeng; HY, Heyuan; GZ, Guzhu; HZ, Huizhou; QT, Qiaotou*.

c *Water samples in September; XFW, Xinfeng; HYW, Heyuan; GZW, Guzhu; HZW, Huizhou; QTW, Qiaotou*.

d *Distance, the distance between adjacent sampling sites*.

### Diversity and richness analysis of bacterial communities

A total of 192,008 raw reads were yielded by pyrosequencing of 16S rRNA gene amplicons, and 138,579 post-trimming reads were obtained. The number of per sample reads varied from 3,806 to 10,981, with an average number of 5,774. OTUs were grouped at the 97% cutoff, and diversity indexes and richness estimates were calculated for each water sample, forming a total of 7, 904 OTUs (Table S1). OTUs 1601–2754 were variously distributed in all samples which were close to the richness estimates of the overall community (ACE and Chaol indicators), suggesting a high diversity of planktonic bacteria in the river. Water samples in the two seasons exhibited a similar level of diversity based on both Shannon and Simpson indexes. The average Shannon indices were, respectively 9.53 and 9.42 for dry and wet seasons. Moreover, the numbers of OTUs or Shannon diversity indices between the dry and wet seasons did not show obvious differences (*P* > 0.05). Although, the microbial community richness of the sampled water has not yet reached saturation at the current sequencing depth (Figures S1A,B), the coverage ranging from 76.48 to 90.16% indicated that the majority of bacterial members had been successfully sampled in all the respective water samples.

### Comparisons of community structures

**Figure 2:**
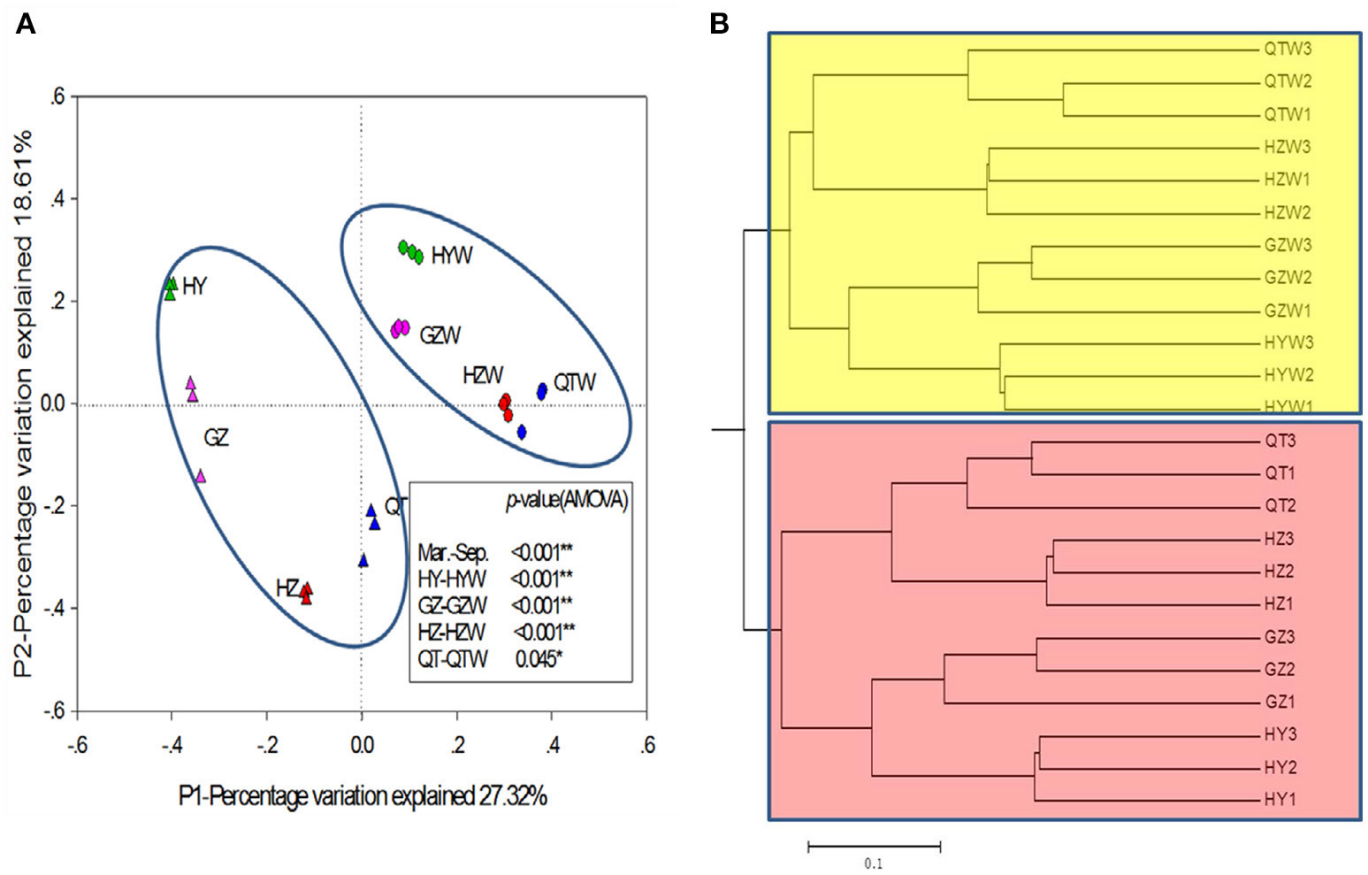
Comparisons of bacterial community structures from the different sampling sites between dry and wet seasons were interrogated using principal coordinate analysis (PCoA, **A**) and clustering analysis of the Bray-curtis distance matrix **(B)**. Water samples in March; XF, Xinfeng; HY, Heyuan; GZ, Guzhu; HZ, Huizhou; QT, Qiaotou. Water samples in September; XFW, Xinfeng; HYW, Heyuan; GZW, Guzhu; HZW, Huizhou; QTW, Qiaotou.

A non-metric multidimensional scaling (NMDS) plot in two-dimensional taxon space based on Bray-Curtis similarities was first used to estimate the overall community resemblance of samples from two seasons. The stress value was 0.08, and the addition of a third dimension did not substantially improve the model. Dry and wet season clusters were distinct when between-sample differences in bacterial community composition were visualized by NMDS ordination (Figure S2). Using ANOISM, Adonis, and MRPP (Zhou et al., 2011) based on the relative abundance of all OTUs detected in dry, and wet seasons, we observed significant differences between the two season clusters (Table S2), and such significances (*p* < 0.05) were also observed at the phylum level, including *Acidobacteria, Actinobacteria, Armatimonadetes, Bacteroidetes, Cyanobacteria, Firmicutes, Planctomycetes, Proteobacteria, Verrucomicrobia* (Table S3). In addition, the principal Coordinate analysis (PCoA) based on the Bray-Curtis distance was used to compare the overall community dissimilarity with relative abundances of OTUs. The water samples of wet season were grouped together and segregated obviously from those of dry season along the P1 (*P* < 0.001), which explained 27.32% of the total variance (Figure 2A). Additionally, the seasonal changes of bacterioplankton populations at each sampling site were also significant (*P* < 0.05). Moreover, separations of community structures between wet and dry seasons were observed based on clustering analysis of the Bray-Curtis distance matrix (Figure 2B).

Hierarchical clustering analysis of the overall microbial communities showed an obvious separation of two broad groups between dry and wet seasons (Figure S3). Moreover, the communities in the upstream sampling sites were well-grouped together, and separated from those in the downstream sites in both seasons. Such clustering patterns are consistent with clustering analysis of river-water physicochemical factors (Figure 1). Eight major OTU groups were well-associated with sampling sites (Figure S3). In groups 2, 4, 5, and 7 in the heatmap, the relative abundance of sequences recovered from GZ, HZ, QT, and HY in dry season was much higher than those from wet season, whereas groups 1, 3, 6, and 8 were mostly contributed by the communities from HZW, HYW, GZW, and QTW in wet season. *Burkholderiales* (3.6, 6.1, 4.9, 5.0, and 3.0%) from *Betaproteobacteria* were the most dominant order in groups 1, 2, 3, 4, 6, while *Actinomycetales* (3.3 and 1.3%) from *Actinobacteria* dominated groups 5 and 8. In addition, *Sphingobacteriales* (1.4%) from class *Sphingobacteria*, and *Flavobacteriales* (2.5%) from class *Flavobacteria* were also more abundant than other populations in groups 6 and 7, respectively. These results suggest that the overall microbial communities along the Dongjiang River display obviously seasonal alteration and the discrepancy of community composition between different sampling sites may be affected by water quality.

### Comparison of community composition based on taxonomy

**Figure 3:**
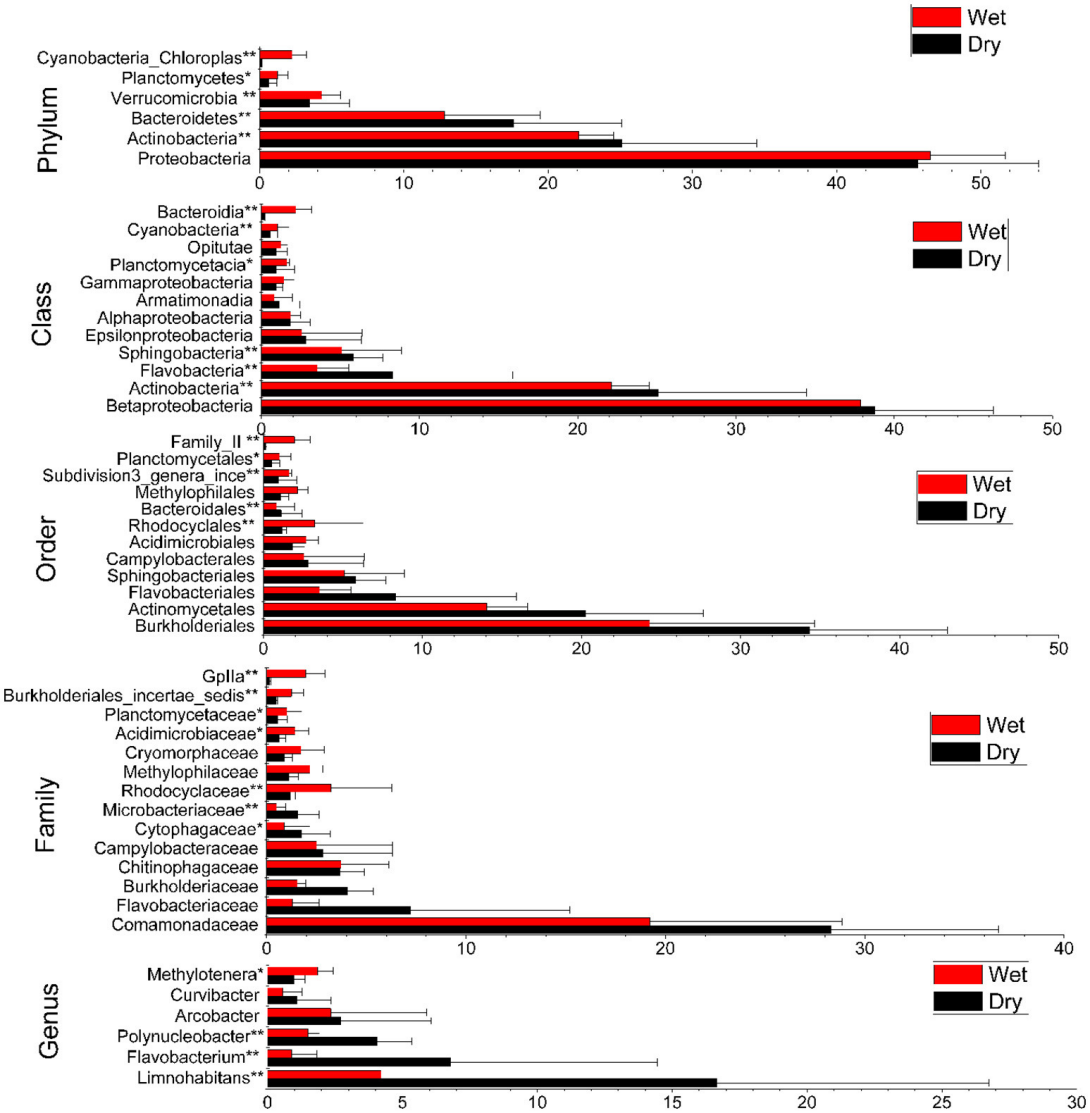
Comparisons of bacterioplankton taxonomy profiles of the dry and wet seasons based on the relative abundance of OTUs. Comparisons were performed at levels of Phylum, Class, Order, Family, and Genus. The most frequently detected taxa (above 1% of relative abundance) in each level are shown. Means of the relative abundance for each taxon at each taxonomical level between the two seasons are compared (^*^p < 0.05, ^**^p < 0.01; mean ± s.e.m).

Most phylotypes of bacterioplankton populations in the Dongjiang River were detected in both dry and wet seasons, with only a few detected at one season (Table S4). The most frequently detected taxa with >1% relative abundance at different classification levels in any season were further analyzed below (Figure 3).

#### Changes at the phylum level

**Figure 4:**
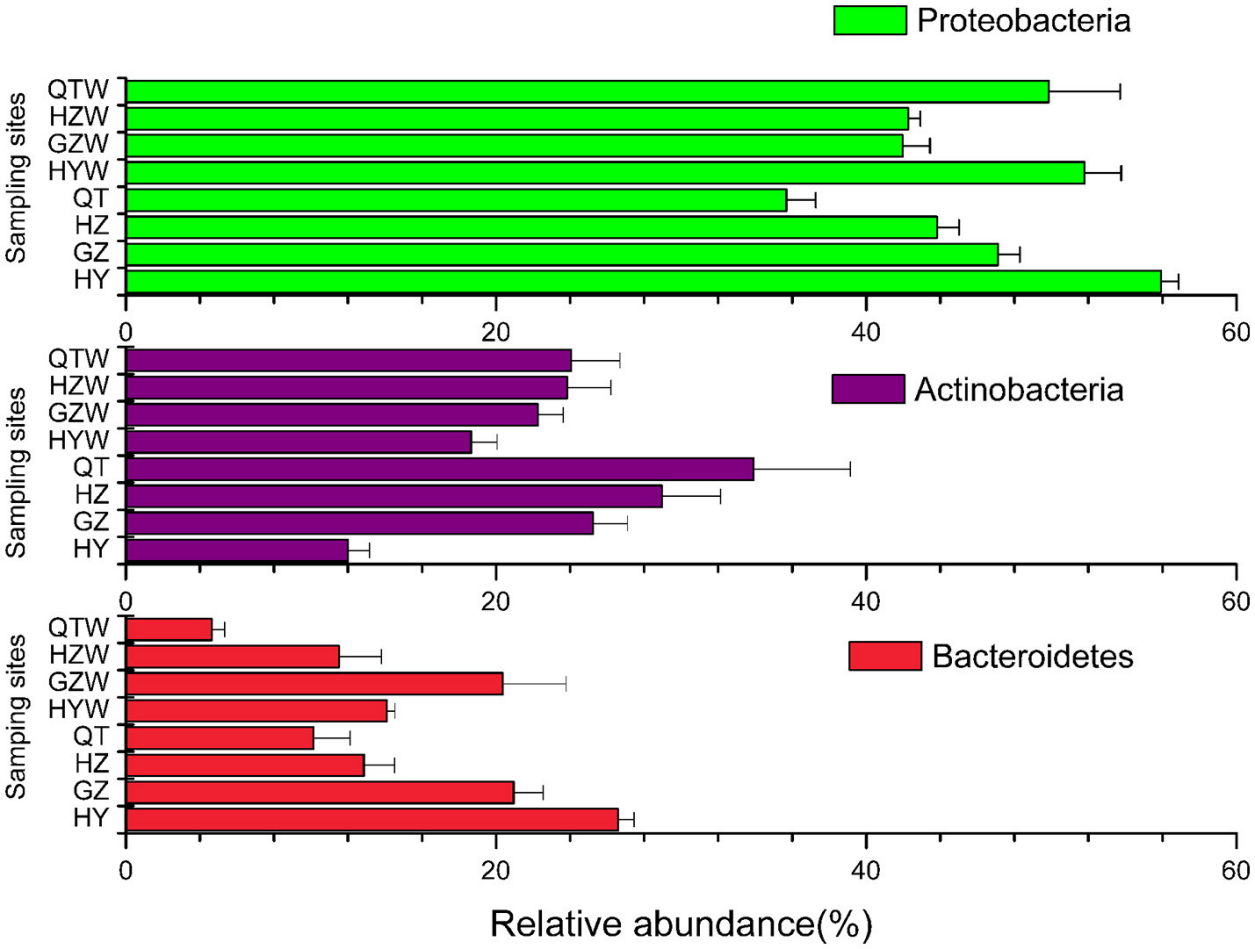
Predominant bacterial phylum in the water column of the Dongjiang River. Over 75% of the organismal diversity in each sample from dry or wet seasons was contributed by the three phyla *Proteobacteria, Actinobacteria*, and *Bacteroidetes*. However, variations in their relative abundance between these two seasons were detected (See Section Results). Water samples in March; XF, Xinfeng; HY, Heyuan; GZ, Guzhu; HZ, Huizhou; QT, Qiaotou. Water samples in September; XFW, Xinfeng; HYW, Heyuan; GZW, Guzhu; HZW, Huizhou; QTW, Qiaotou.

Across all water samples we detected 18 bacterial phyla, six of which were abundant (relative abundance higher than 1% at the phylum level in both seasons; Figure 3). Most sequences (84.83%) were members of three phyla: *Proteobacteria* (45.72%), *Actinobacteria* (24.55%), and *Bacteroidetes* (14.56%). Strikingly, *Actinobacteria* and *Bacteroidetes* seemed to be more season-associated, whose relative abundances were obviously higher in dry season (*P* < 0.01), while that of *Proteobacteria* was slightly lower in dry season than in wet season (Figure 3). On the other hand, considering that over 75% of the organismal diversity in each sample from dry or wet season was contributed by the above three dominant phyla, the variations of their relative abundances between these two seasons at each sampling site were also detected. The relative abundances of *Actinobacteria* obviously increased from upstream to downstream sites along the river in both seasons, while only a decrease of those of *Proteobacteria* and *Bacteroidetes* in dry season was observed (Figure 4).

#### Changes at the class level

A total of 40 classes were detected with 12 being abundant (relative abundance >1% at the class level at both seasons). The relative abundance of *Betaproteobacteria* was the highest, but no significant difference between two seasons was observed (Figure 3). *Actinobacteria, Flavobacteria*, and *Sphingobacteria* ranked the 2nd, 3rd, and 4th abundant classes detected, whose relative abundances obviously decreased from dry season to wet season, while *Planctomycetacia, Cyanbacteria*, and *Bacteriodia* significantly increased (*P* < 0.05).

#### Changes at the genus level

A total of 186 genera were detected from the four sampling sites in two seasons. Thirty five were found to be differentially distributed between seasons by the response ration method, among which 25 were significantly more abundant, and the rest 10 were statistically less abundant (*P* < 0.05) in the dry season than in the wet season. Additionally, *Limnohabitans, Flavobacterium, Polynucleobacter, Arcobacter, Curvibacter*, and *Methylotenera* were the dominant genera with individual relative abundances higher than 1% in at least one season. Moreover, the former three dominated the dry season (*P* < 0.01, Figure 3).

#### OTU-level comparisons of seasonal changes

The most OTUs detected were members of *Proteobacteria* (3,962; 50.13%), followed by *Bacteroidetes* (1,345; 17.02%), and *Actinobacteria* (1,204; 15.23%; Table S5). Among the total 7,904 OTUs in all samples, 2,730 (34.54%) were shared by these two seasonal samples (common), while 2,827 (35.77%) and 2,347 (29.69%) were present only in the dry season or the wet season samples (unique). For those common OTUs, 395 decreased significantly (*P* < 0.05), and 440 increased significantly (*P* < 0.05) in the wet season compared with the dry season (Table S6). For those unique ones, most of them were members of the most abundant phyla (Table S6), with 1,463 and 1,141 detected for *Proteobacteria* at dry, and wet seasons, respectively, and 543 and 376 for *Bacteroidetes*. Therefore, as defined by OTUs that reached species levels, the seasonal variations of the bacterioplankton communities in the Dongjiang River were obvious.

To further compare the difference of planktonic bacteria between dry and wet seasons, the phylotypes with at least six OTUs detected among all samples were identified to conduct response ratio analysis based on the relative abundance of all OTUs (He et al., 2012). OTUs with significantly seasonal changes mainly distributed in the most abundant phyla, including *Proteobacteria, Bacteroidetes*, and *Actinobacteria* (Figure S4). The relatively high OTU number of some family, such as *Comamonadaceae* of *Betaproteobacteria*, changed significantly between the two seasons (*P* < 0.05). These results suggested that significant changes between dry and wet seasons occurred to the most abundant phylotypes, and some specific planktonic bacteria dynamically responded to the seasonal change.

### Relationships between the bacterioplankton community structure and physicochemical parameters

Patterns of dynamic changes in bacterioplankton communities along the Dongjiang River would be affected by the fluctuations of physicochemical parameters of water columns. Based on the forward selection of the significant environmental factors and the variance inflation factors (VIF) of the selected factors <20, redundancy analysis (RDA) was conducted to correlate the difference of bacterioplankton communities at different taxonomic levels (from phylum to OTU) with the possibly key environmental factors (Table 2). At the phylum level, DIN, TSS, and temperature were identified as the three crucial factors, which contributed to 72.7% of total variation for the bacterioplankton community with 59.6 and 11.7% by the first, and second axes respectively. At the class level, TN, TSS, and temperature took the major responsibility (65.7%) for reshaping the microbial communities, with 55.9% by the first axis and 8.3% by the second axis. At the order level, the top three environmental factors were ${\text{NH}}_{4}^{+}$, ${\text{NO}}_{3}^{-}$, and TOC, which accounted for 74.9% of the total variation of the bacterioplankton community, with 65.2% by the first axis, and 7.9% by the second axis. At the family and genus levels, the top contributing factors were identified as ${\text{NO}}_{3}^{-}$, DIN, and temperature, which contributed 73.1 and 68.4%, respectively to the bacterioplankton distribution, with majority at first axis. Finally, ${\text{NH}}_{4}^{+}$, TOC, and temperature explained 46.8% (*p* < 0.01) for the distribution at the OTU level. These results suggested that the nitrogen nutrients, TOC, and temperature closely correlated with the seasonal change, and were the dominant factors affecting the distribution of bacterioplankton in the river, although some different environmental factors were also identified to significantly explain the community differentiation at other taxonomic levels.

**Table 2:** Redundancy analyses of the relationship between the bacterioplankton communities at different taxonomic levels and physicochemical water properties.

**Level**	**The three top highest-loading water properties**^**a**^			**First axis (%)**	**Second axis (%)**	**Total explanation (%)**	**F-ratio^b^**	***p*^b^**	***r*^c^**	***p*^c^**
OTU	${\text{NH}}_{4}^{+}$	TOC	Tem	25.0	13.0	46.8	5.87	0.005	0.692	0.001
Genus	${\text{NO}}_{3}^{-}$	DIN	Tem	66.3	1.7	68.4	14.41	0.005	0.391	0.003
Family	${\text{NO}}_{3}^{-}$	DIN	Tem	69.5	2.1	73.1	19.10	0.005	0.646	0.001
Order	${\text{NH}}_{4}^{+}$	${\text{NO}}_{3}^{-}$	TOC	65.2	7.9	74.9	19.91	0.005	0.516	0.001
Class	TN	TSS	Tem	55.9	8.3	65.7	12.75	0.005	0.412	0.001
Phylum	DIN	TSS	Tem	59.6	11.7	72.7	17.74	0.005	0.627	0.001

a *Variables selected by forward selection based VIF with 999 Monte Carlo permutations*.

b *F- and p-values of analysis of variance*.

c *Mantel analyses for the relationship between bacterioplankton communities at different levels and selected environmental factors*.

Mantel tests were conducted to correlate the bacterioplankton structure with the water qualities. Three physical (pH, TSS, and temperature) and three chemical (${\text{NH}}_{4}^{+}$-N, ${\text{NO}}_{3}^{-}$-N, and TOC) parameters were initially selected by the BioEnv procedure, which exhibited the highest Pearson correlation with bacterioplankton communities. Then, partial Mantel tests were performed to link the bacterioplankton communities estimated by the relative abundance of all detected OTUs to the physical and chemical parameters as selected above. Overall, specific bacterioplankton communities at different taxonomic levels (phylum, class, order, family, and genus) were significantly associated with the selected physical and chemical parameters (*P* < 0.05 and < 0.01; Tables S7–S11). For example, at the phylum level, most members of the phyla *Acidobacteria, Armatimonadetes, Nitrospira, Proteobacteria, WS3, Cyanobacteria, Gemmatimonadetes*, and *Firmicutes* significantly (*P* < 0.01) correlated with selected chemical properties, *Bacteroidetes*, and *Verrucomicrobia* (*P* < 0.01) with selected physical properties, while *Actinobacteria*, and *Planctomycetes* (*P* < 0.01 and *P* < 0.05) with both. In addition, such patterns were also discovered in a certain number of unclassified phylotypes at different taxonomic levels, suggesting that taxonomically undetermined bacterioplankton might be as well-shaped by the water physiochemical parameters (Tables S8–S11). In summary, the bacterioplankton communities are affected by the seasonally changing physicochemical factors of the water column in the Dongjiang River.

In order to further identify the key environmental factors responsible for the seasonal change of bacterioplankton communities, the relationship between the relative abundances of dominant bacterial groups (average above 1% at different classification levels in one season), and water properties were analyzed by Linear regressions using SPSS 17.0. Among the effects of different physicochemical factors (such as ${\text{NH}}_{4}^{+}$-N, ${\text{NO}}_{3}^{-}$-N, TOC, pH, TSS, and temperature) upon the specific phylotypes at different taxonomic levels, the effects of ${\text{NO}}_{3}^{-}$-N, and temperature were broad. For example, the relative abundances of the dominant bacterial phyla (e.g., *Bacteroidetes, Cyanobacteria, Planctomycetes*), classes (e.g., *Gammaproteobacteria, Bacteroidia, Cyanobacteria*), and orders (e.g., *Burkholderiales, Rhodocyclales, Methylophilales*) all significantly correlated across the ${\text{NO}}_{3}^{-}$-N content and/or the temperature gradients, with either a positive correlation (e.g., the phylum *Planctomycetes* with ${\text{NO}}_{3}^{-}$-N gradient, the order *Methylophilales* with temperature), or a negative one (e.g., the phylum *Bacteroidetes with* ${\text{NO}}_{3}^{-}$-N gradient, the order *Burkholderiales* with temperature; Figures S5, S6). Additionally, the contents of ${\text{NH}}_{4}^{+}$-N showed positive correlations with the relatively abundant classes (e.g., *Alphaproteobacteria* and *Actinobacteria*), while TOC and TSS showed negative and positive correlations with *Bacteroidia* and *Actinobacteria*, respectively (Figure S7). Overall, bacterioplankton phylotypes correlating with the ${\text{NO}}_{3}^{-}$-N and temperature covered broader levels of taxonomic resolution than those with ${\text{NH}}_{4}^{+}$-N, TOC, and TSS, indicating that ${\text{NO}}_{3}^{-}$-N content, and temperature were more likely the determinant than ${\text{NH}}_{4}^{+}$-N, TOC, and TSS when it comes to the shaping of bacterioplankton communities across the Dongjiang River.

## Discussion

Large rivers are dominant features of the landscape, profoundly responsible for regulating carbon, and nitrogen cycles within the local ecology. The Dongjiang River, with 90% drainage area in the Guangdong province (Liu et al., 2010), is not only crucial to the sustainable development of the Pearl River Delta, but also serves as the main drinking water source for Hongkong. Bacterioplankton play an important role in river biogeochemical processes, which affect the water qualities. However, current information depicting temporal, and special changes in microbial community structure in large rivers is limited. The primary goal of this present study was to understand the patterns in which bacterioplankton respond to those changes during dry and wet seasons along the Dongjiang River, which provided a valuable opportunity to study the influences of typical seasonal change on biogeochemical cycles in such systems. Overall, our results demonstrated that seasonality strongly impacted the bacterioplankton communities in the Dongjiang River (Figure 2, Figure S2), which were highly sensitive to the fluctuations of temperature and water physiochemical properties.

Dynamic seasonal changes seem to be a common feature of bacterioplankton communities in various aquatic ecosystems. In the Dongjiang River, bacterioplankton communities of dry season were sampled during the spring freshet, and were always collected at a low temperature, and river flow, while those of wet season were sampled in the late summer at a warm temperature and a fast river flow. According to PCoA, individual communities from all sampling sites exhibited significant seasonal changes (Figure 2A), and undoubtedly, so did the overall communities. The environmental conditions shared by the four sampling sites along the river clearly separated into dry and wet seasons (Figure 1). The bacterioplankton communities distributed consistently with the classification of above conditions (Figure 1, Figure S3), which suggested their strong correlation. Similarly, the river microbial communities themselves were reported to have a seasonal cycle, which maintained a stable state from one season to another (Crump and Hobbie, 2005; Crump et al., 2009). In lakes, distinct communities were present in different seasons, which usually resulted from major seasonal events such as lake mixing, temperature, and productivity (Kara et al., 2013; Wilhelm et al., 2014). Additionally, the bacterial community at a temperate marine coastal site off Plymouth, UK showed strong periodic seasonal patterns, and the day length seemed to be important in regulating them (Gilbert et al., 2012).

*Proteobacteria* were the predominant group with *Betaproteobacteria* being the most abundant class in the Dongjiang River. Previous efforts on the Dongjiang River suggested that *Betaproteobacteria* were always a major component of the microbial community and may well-adapt to most freshwater environments (Liu et al., 2012). It have been confirmed to be one of the typical, dominant, and important freshwater bacterial assemblages in aquatic habitats including rivers (e.g., the Elbe River, the Spittelwasser River, the Ipswich, Parker Rivers), and lakes (e.g., Crystal Bog Lake, Lake Zurich) (Brummer et al., 2003; Crump and Hobbie, 2005; Newton et al., 2006; Crump et al., 2009; Salcher et al., 2013). The relative abundance of *Alphaproteobacteria* was higher than *Gammaproteobacteria* in both dry and wet seasons, which was inconsistent with previous reports where these two classes were in equal proportions (Liu et al., 2012). This slight difference may be caused by random errors, such as the different sequencing depths between clone libraries and 454 pyrosequencing. It is reported that *Betaproteobacteria* are often the numerically dominant group in freshwater environments but are in relatively low abundance in the ocean (Newton et al., 2011). *Alphaproteobacteria* and *Gammaproteobacteria* as the most abundant classes constitute the bacterioplankton community and prefer saltwater (Biers et al., 2009; Teeling et al., 2012; Wemheuer et al., 2014), although they were widely distributed in rivers, and lakes all over the globe (Crump and Hobbie, 2005; Newton et al., 2011; Liu et al., 2012). Our hypothesis is, members of the above classes present in freshwater systems are very likely the transient passengers brought by the surroundings, or they are simply not the same clusters to those present mainly in seawater. Many bacteria in the *Proteobacteria* are particle-associated and play important roles in aquatic carbon and nitrogen cycling (Dang and Lovell, 2016). However, the *Proteobacteria* appeared to be comprised more from free living bacterioplankton (33.4%) than from particles (11.6%), and *Alphaproteobacteria* were the most dominant subphylum of *Proteobacteria* in the free-living samples in large rivers of the Mississippi River Basin (Jackson et al., 2014). The balance between particle-associated and free living *Proteobacteria* seems to be an evolutionary adaptation to marine or river environments. It is really worth further exploring and verifying free-living and particle-associated bacterioplankton in the Dongjiang River, because their compositions and eco-functions may differ (Dang and Lovell, 2016).

Among abundant freshwater *Betaproteobacteria*, the genus *Limnohabitans* were the dominant group in the Dongjiang River. Reports of *Limnohabitans* detected in marine and terrestrial systems are scarce, thus their natural distribution seems to be restricted to freshwater systems (Hahn et al., 2010; Kasalicky et al., 2013). Moreover, the *Limnohabitans* were reported to have a prominent role in freshwater bacterioplankton communities due to their high rates of growth, and substrate uptake, high mortality rates and utilization of algal exudates (Jezbera et al., 2013; Kasalicky et al., 2013). The members of the family *Enterobacteriaceae*, order *Enterobacteriales*, class *Gammaproteobacteria*, were often used as indicator to track pollutants in surface waters (Stoeckel and Harwood, 2007), and they were not detected in the Dongjiang River, suggesting that the overall river is kept in good water quality with effective pollution control. Notably, the largest seasonal changes of bacterioplankton communities in OTU levels were found in the phylum *Proteobacteria* (Figure S4), suggesting its high sensitivity to seasonal environment changes.

*Actinobacteria* were identified as the second abundant group in the Dongjiang River. Historically, *Actinobacteria* were considered the primarily active groups resident in soils (Goodfellow and Williams, 1983). This notion has been refreshing with more and more studies of aquatic systems. In fact, the *Actinobacteria* are common seen, and often the numerically dominant group in various freshwater ecosystems (Crump and Hobbie, 2005; Allgaier et al., 2007; Wilhelm et al., 2014). Moreover, several studies have documented that the abundance of *Actinobacteria* in freshwater lakes exhibited seasonal dependence (e.g., spring–summer; Allgaier et al., 2007; Wilhelm et al., 2014). What we discovered in the present study reconciles with that case, in which *Actinobacteria* were in much higher abundance in the dry season than in the wet season (Figure 3), highlighting the potential linkage between the biological communities in the Dongjiang River, and the seasonal environmental factors. Overall, *Actinobacteria* may well-adapt to various freshwater ecosystems and dynamic seasonal changes due to its pronounced ecophysiological plasticity (Allgaier et al., 2007).

*Bacteroidetes* were the third abundant phylum in the Dongjiang River, consistent with the findings that it has been one of the most common phyla recovered from lake epilimnion (the upper layer of water in a lake), and rivers (Crump and Hobbie, 2005; Newton et al., 2011). However, unlike other common freshwater groups, it does not exhibit any freshwater-specificity, and has been spotted in broad environments, such as soil, ocean, or as symbionts within humans, animals, and plants (Newton et al., 2011). Moreover, the existent patterns of *Bacteroidetes* in similar environments were different sometimes. For example, the low abundance of *Bacteroidetes* was reported in southern region of North Sea (Wemheuer et al., 2014), while this phylum in bacterioplankton communities was dominating the Southern Ocean (Jamieson et al., 2012). Enormous phenotypic and metabolic diversities of *Bacteroidetes* phylum may well-account for its broad adaptions in various environments.

Another striking data are the significant differences of 16S rRNA genes associated with *Cyanobacteria* between dry, and wet seasons, whose relative abundance was about 10-fold higher in wet season relative to dry season (Figure 3). Similar observations were also reported in Laurentian Great Lakes where *Cyanobacteria* were two orders of magnitude higher in summer (10^5^–10^6^ cells mL^−1^) than winter months (2.6–3.7 × 10^3^ cells mL^−1^) using direct epifluorescence enumerations (Matteson et al., 2011). The abundant distributions of *Cyanobacteria* were also discovered across many lakes using both optical and molecular technique (Carrick and Schelske, 1997; Wilhelm et al., 2006). Wilhelm reported recently that sequences associated with *Cyanobacteria* dominated summer samples at all stations of Lake Erie (Wilhelm et al., 2014). Therefore, *Cyanobacteria* in freshwater ecosystems exhibited seasonally changing patterns, with higher abundance in warm seasons, which suggests that temperature may be the key factor for its distribution. The linear regression analysis further confirmed the significant relationship between them.

In the Dongjiang River, bacterioplankton communities were strongly correlated with environmental variables (Table 2). The best combinations of environmental variables for describing variability in bacterioplankton communities were inconsistent at different taxonomic levels, which was not a surprise because of a possible high degree of covariation among these parameters. However, most of these RDA models included factors describing the physical properties (temperature and TSS) and the concentrations of chemical variables (${\text{NH}}_{4}^{+}$-N, ${\text{NO}}_{3}^{-}$-N, DIN, and TOC) of the river water. Further mantel tests and linear regression analyses identified temperature and ${\text{NO}}_{3}^{-}$-N as more important influential factors than others on shaping the bacterioplankton communities (Figures S5, S6). Water temperature was previously reported to be the strongest predictor of changes in communities of the Ipswich and Parker Rivers, demonstrating the strong seasonality of river bacterioplankton communities (Crump and Hobbie, 2005). The bacterioplankton perform important ecological functions, such as regenerating nutrients, decomposing organic matter, and participating in the base of microbial food webs, all of which are regulated by the temperature (Adams et al., 2010). Therefore, the obviously different temperatures between dry, and wet seasons in the Dongjiang River played an important role in modulating the compositions, and structures of bacterioplankton communities.

Nitrogen nutrients especially nitrate were also identified as important factors influencing the bacterioplankton community structure. This was consistent with our previous studies in the Dongjiang River that community structure and composition of bacterioplankton mainly responded to fluctuations of nitrogen nutrients (Liu et al., 2012). Additionally, the concentrations of ${\text{NO}}_{3}^{-}$-N were also considered one of the key variables to account for most of the variability in bacterial community composition of the six largest rivers of the pan-arctic watershed (Crump et al., 2009). Overall, these environmental variables correlating with seasonal variability could contribute a large proportion of the variability to bacterioplankton communities along the river, since no single factor could convincingly explain the complex dynamic patterns.

Large rivers are often characterized by high loads of TSS (Jackson et al., 2014), which is mainly detritus from amorphous aggregations of suspended sediments with many heterotrophic benthic bacteria attaching to Tang et al. (2010). TSS was not the most important factor for shaping the bacterioplankton seasonality in the present study, possibly due to its insignificant seasonal changes (*p* > 0.05). Our data however, revealed a potential positive correlation between TSS, and the two groups (phylum *Actinobacteria* and order *Actinomycetales*; Figure S7). The lotic and dynamic nature of large rivers present an interesting situation in that allochthonous terrestrial inputs were pouring into water column and river sediments through runoff and they also affected the TSS concentration, which contribute to the transport of terrestrial microorganisms in the river ecosystems. Therefore, it is not surprise that TSS was one of the environmental factors affecting the distribution of bacterioplankton. Additionally, TSS in rivers are excellent sites for bacterial adsorption, and production, because they possess much of the nutrient or organic matter and may develop a distinct bacterial community compared with the free-living bacterioplankton (Ochs et al., 2010; Jackson et al., 2014). TSS were reported as the most significant environmental factor balancing the connectivity between free-living and particle-associated bacterial communities in Lake Taihu (Tang et al., 2015). More samples from the Dongjiang River are needed to explore the mechanism of TSS on the distribution of bacterioplankton and to understand the dynamics and function of free-living and particle-associated bacterioplankton in large river ecosystem.

## Conclusions

This study focused on effects of seasonality on the diversity and composition of bacterioplankton in the Dongjiang River served as drinking water source for Hongkong. In conclusion, high-throughput pyrosequencing revealed higher diversities of bacterioplankton at different taxonomic levels in the Dongjiang River compared with the previous clone libraries (seven phyla) (Liu et al., 2012). This study demonstrated obvious seasonal effects on the composition of bacterioplankton communities in the Dongjiang River, and showed a common, globally distributed set of freshwater bacterial populations. Water temperature and nitrogen nutrients (especially ${\text{NO}}_{3}^{-}$ concentration) were identified as important factors affecting the seasonal distribution. This study pointed out the need to supplement the current data with a functional analysis (e.g., metatranscriptomes or metaproteomes) to better understand the seasonal changes in the physiology of individual taxa, which enables to profile the seasonal transitions in ecological functions of bacterioplankton communities. What's more, it would be necessary to expand seasonal samplings in large river ecosystems serving as drinking water sources like the Dongjiang River, since obviously important microbial and geochemical processes that have been missed previously are occurring. Additionally, it is imperative to explore the compositional differences in particle-associated and free-living microbial assemblages in large river systems, because their composition and eco-functions may largely differ from each other (Dang and Lovell, 2016).

## Author contributions

JG and MX initiated and designed the research; WS and CX collected samples and performed the research; WS and MX analyzed microbial diversity and performed the statistical analysis; WS and MX analyzed data; WS, MX, JG, and GS wrote the manuscript. All authors reviewed the manuscript.

### Conflict of interest statement

The authors declare that the research was conducted in the absence of any commercial or financial relationships that could be construed as a potential conflict of interest.
