# Supplementary material for: Seasonality Affects the Diversity and Composition of Bacterioplankton Communities in Dongjiang River, a Drinking Water Source of Hong Kong
Source: Front Microbiol. 2017 Aug 31;8:1644. doi: 10.3389/fmicb.2017.01644 (PMC5583224; doi:10.3389/fmicb.2017.01644)
Supplement: Supplementary file 1 [file Table1.DOCX]

**Table S1** Estimates of phylotype coverage and diversity estimation of the 16S rRNA gene from the pyrosequencing analysis

|  | Sample  sites |  | | | OTUs number | Alpha diversity | | | | | | |
| --- | --- | --- | --- | --- | --- | --- | --- | --- | --- | --- | --- | --- |
| Time |  | Raw reads | Raw analyzed | Coverage |  | Shannon | Simpson | Inverse simpson | Ace | chaol | pielou.  Evenness (J) |  |
| Mar. | HY1 | 5933 | 4605 | 83.84% | 1607 | 6.85 | 0.0022 | 411.54 | 2458.23 | 2437.02 | 0.93 |  |
|  | HY2 | 6552 | 4880 | 83.22% | 1715 | 6.91 | 0.0022 | 422.06 | 2763.04 | 2666.62 | 0.93 |  |
|  | HY3 | 6713 | 5111 | 84.72% | 1747 | 6.99 | 0.0015 | 594.78 | 2633.05 | 2572.45 | 0.94 |  |
|  | GZ1 | 9161 | 6348 | 88.11% | 1738 | 6.81 | 0.0025 | 382.51 | 2624.41 | 2624.71 | 0.91 |  |
|  | GZ2 | 7449 | 5454 | 84.60% | 1905 | 7.10 | 0.0013 | 675.53 | 2838.95 | 2742.01 | 0.94 |  |
|  | GZ3 | 7232 | 5346 | 83.93% | 1902 | 7.11 | 0.0013 | 673.61 | 2910.69 | 2807.43 | 0.94 |  |
|  | HZ1 | 10781 | 8160 | 86.24% | 2535 | 7.28 | 0.0012 | 751.04 | 3840.84 | 3735.01 | 0.93 |  |
|  | HZ2 | 13151 | 9849 | 88.67% | 2683 | 7.25 | 0.0014 | 665.69 | 3946.21 | 3770.71 | 0.92 |  |
|  | HZ3 | 15106 | 10981 | 90.16% | 2754 | 7.29 | 0.0013 | 715.89 | 3874.26 | 3909.92 | 0.92 |  |
|  | QT1 | 8698 | 6174 | 86.09% | 1969 | 7.09 | 0.0013 | 683.79 | 2986.72 | 2865.62 | 0.94 |  |
|  | QT2 | 7862 | 5553 | 86.01% | 1689 | 6.86 | 0.0018 | 506.21 | 2678.06 | 2616.62 | 0.92 |  |
|  | QT3 | 8649 | 6214 | 85.82% | 1981 | 7.07 | 0.0014 | 644.79 | 3067.14 | 2870.08 | 0.93 |  |
| Dry | Total | 107287 | 78675 |  | 5557 | 9.53 |  |  |  |  |  |  |
| Sep. | HYW1 | 6402 | 4697 | 81.18% | 1820 | 7.07 | 0.0013 | 654.01 | 2945.58 | 2762.72 | 0.94 |  |
|  | HYW2 | 5589 | 3891 | 76.48% | 1741 | 7.11 | 0.0010 | 775.65 | 2978.53 | 2773.48 | 0.95 |  |
|  | HYW3 | 6609 | 4400 | 79.73% | 1830 | 7.15 | 0.0010 | 813.92 | 2969.89 | 2756.31 | 0.95 |  |
|  | GZW1 | 5484 | 3812 | 77.31% | 1674 | 7.07 | 0.0011 | 733.31 | 2850.94 | 2617.64 | 0.95 |  |
|  | GZW2 | 7418 | 5284 | 83.25% | 1926 | 7.15 | 0.0011 | 784.86 | 2963.94 | 2866.31 | 0.95 |  |
|  | GZW3 | 7300 | 4968 | 81.88% | 1895 | 7.14 | 0.0011 | 764.69 | 2959.09 | 2934.97 | 0.95 |  |
|  | HZW1 | 5737 | 3806 | 77.61% | 1646 | 7.06 | 0.0010 | 785.81 | 2806.01 | 2653.02 | 0.95 |  |
|  | HZW2 | 9031 | 5606 | 85.94% | 1852 | 7.09 | 0.0012 | 729.04 | 2765.98 | 2606.45 | 0.94 |  |
|  | HZW3 | 5489 | 3778 | 77.90% | 1633 | 7.06 | 0.0010 | 785.54 | 2802.80 | 2554.15 | 0.95 |  |
|  | QTW1 | 7854 | 6430 | 89.36% | 1601 | 6.60 | 0.0031 | 306.01 | 2397.52 | 2315.33 | 0.89 |  |
|  | QTW2 | 9264 | 7193 | 89.77% | 1737 | 6.70 | 0.0028 | 345.79 | 2589.72 | 2618.04 | 0.90 |  |
|  | QTW3 | 8544 | 6039 | 88.49% | 1610 | 6.76 | 0.0022 | 429.84 | 2442.63 | 2413.88 | 0.92 |  |
| Wet | total | 84721 | 59904 |  | 5077 | 9.42 |  |  |  |  |  |  |
|  | Total | 192008 | 138579 |  |  |  |  |  |  |  |  |  |
|  | Average |  | 5774 | 84.18% |  |  |  |  |  |  |  |  |
