# Supplementary material for: Seasonality Affects the Diversity and Composition of Bacterioplankton Communities in Dongjiang River, a Drinking Water Source of Hong Kong
Source: Front Microbiol. 2017 Aug 31;8:1644. doi: 10.3389/fmicb.2017.01644 (PMC5583224; doi:10.3389/fmicb.2017.01644)
Supplement: Supplementary file 2 [file Table2.DOCX]

**Table S2** Significance tests of the overall microbial community structure of the dry and wet seasons with three different statistical approaches

| Pair of samples compared | Adonis^a^ | | ANOSIM^b^ | | MRPP^c^ | |
| --- | --- | --- | --- | --- | --- | --- |
|  | F | *p* | R | *p* | δ | *p* |
| Dry season-Wet season | 6.733 | **0.001** | 0.694 | **0.001** | 0.631 | **0.001** |

^a^adonis: Analysis of variance using distance matrices, ^b^anonim: Analysis of similarities, ^c^MRPP: Multiple Response Permutation Procedure. All three tests are non-parametric multivariate analyses based on dissimilarities among samples. P-value of corresponding significance test. The values in bold indicate the significant difference between the dry and wet seasons (at the levels of *P*<0.01, or *P*<0.05) by the tests.
