# Supplementary material for: Seasonality Affects the Diversity and Composition of Bacterioplankton Communities in Dongjiang River, a Drinking Water Source of Hong Kong
Source: Front Microbiol. 2017 Aug 31;8:1644. doi: 10.3389/fmicb.2017.01644 (PMC5583224; doi:10.3389/fmicb.2017.01644)
Supplement: Supplementary file 3 [file Table3.DOCX]

**Table S3 Statistical analysis of differences in the microbial community composition and structure between the dry and wet seasons at the community and phylum levels.**

|  |  | MRPP^a^ | | anosim^b^ | | adonis^c^ | |
| --- | --- | --- | --- | --- | --- | --- | --- |
|  | No. OTUs | δ | *p* | R | *p* | R^2^ | *p* |
| All phylotypes | 7904 | 0.624 | 0.001 | 0.702 | 0.001 | 0.237 | 0.001 |
| Acidobacteria^d^ | 44 | 0.755 | **0.001** | 0.232 | **0.011** | 0.164 | **0.003** |
| Actinobacteria | 1204 | 0.521 | **0.001** | 0.870 | **0.001** | 0.313 | **0.001** |
| Armatimonadetes | 14 | 0.628 | **0.004** | 0.182 | **0.007** | 0.147 | **0.002** |
| Bacteroidetes | 1345 | 0.704 | **0.001** | 0.515 | **0.001** | 0.206 | **0.001** |
| Chloroflexi | 4 | 0.928 | 0.002 | 0.108 | 0.993 | 0.000 | 1.000 |
| Cyanobacteria | 106 | 0.657 | **0.001** | 0.793 | **0.001** | 0.339 | **0.001** |
| Firmicutes | 87 | 0.882 | **0.001** | 0.218 | **0.002** | 0.126 | **0.001** |
| Fusobacteria | 14 | 0.948 | 0.010 | 0.096 | 0.993 | 0.000 | 1.000 |
| Gemmatimonadetes | 16 | 0.853 | 0.001 | 0.030 | 0.767 | 0.000 | 1.000 |
| Nitrospira | 21 | 0.851 | 0.001 | 0.111 | 0.009 | 0.000 | 1.000 |
| OD1 | 5 | 0.936 | 0.007 | 0.036 | 0.035 | 0.000 | 1.000 |
| Planctomycetes | 74 | 0.677 | **0.001** | 0.788 | **0.001** | 0.299 | **0.001** |
| Proteobacteria | 3962 | 0.651 | **0.001** | 0.529 | **0.001** | 0.205 | **0.001** |
| Synergistetes | 4 | 0.946 | 0.023 | 0.108 | 0.992 | 0.000 | 1.000 |
| TM7 | 2 | 0.950 | 0.085 | 0.303 | 1.000 | 0.000 | 1.000 |
| unclassified | 721 | 0.621 | **0.001** | 0.569 | **0.001** | 0.210 | **0.001** |
| Verrucomicrobia | 276 | 0.639 | **0.001** | 0.706 | **0.001** | 0.244 | **0.001** |
| WS3 | 5 | 0.814 | 0.001 | 0.164 | 0.004 | 0.000 | 1.000 |

^a^MRPP: Multiple Response Permutation Procedure

^b^anonim: Analysis of similarities

^c^adonis: Analysis of variance using distance matrices

^d^The red font denote significance of MRPP, anonim and adonis.
