# Supplementary material for: Seasonality Affects the Diversity and Composition of Bacterioplankton Communities in Dongjiang River, a Drinking Water Source of Hong Kong
Source: Front Microbiol. 2017 Aug 31;8:1644. doi: 10.3389/fmicb.2017.01644 (PMC5583224; doi:10.3389/fmicb.2017.01644)
Supplement: Supplementary file 4 [file Table4.DOCX]

**Table S4 The classified phylotypes detected at different taxonomical levels**

|  | Domain | Phylum | Class | Order | Family | Genus |
| --- | --- | --- | --- | --- | --- | --- |
| No. detected phylotypes | 1 | 18 | 40 | 66 | 100 | 186 |
| Shared between the dry and wet seasons | 1 | 16 | 32 | 50 | 70 | 100 |
| Only detected in dry season |  | 1 | 2 | 4 | 14 | 54 |
| Only detected in wet season |  | 1 | 6 | 12 | 16 | 32 |
