# Supplementary material for: Seasonality Affects the Diversity and Composition of Bacterioplankton Communities in Dongjiang River, a Drinking Water Source of Hong Kong
Source: Front Microbiol. 2017 Aug 31;8:1644. doi: 10.3389/fmicb.2017.01644 (PMC5583224; doi:10.3389/fmicb.2017.01644)
Supplement: Supplementary file 5 [file Table5.DOCX]

**Table S5 Numbers of OTUs detected between the dry and wet seasons**

|  | No. of OTUs | | | | |  |
| --- | --- | --- | --- | --- | --- | --- |
|  |  | Total (%)^b^ | | Avg^c^ |  |  |
| Phylum | Total (%)^a^ | Dry | Wet | Dry | Wet | *P* (unpaired t test)^d^ |
| Acidobacteria | 44 (0.56) | 19 (0.34) | 39 (0.77) | 5.75 ± 4.31 | 14.67 ± 5.97 | 0.00 |
| **Actinobacteria^e^** | **1204 (15.23)** | **964 (17.35)** | **785 (15.46)** | **436.83 ± 128.62** | **338.83 ± 39.32** | **0.31** |
| Armatimonadetes | 14 (0.18) | 10 (0.18) | 10 (0.20) | 4.67 ± 2.42 | 3.08 ± 1.73 | 0.23 |
| **Bacteroidetes** | **1345 (17.02)** | **969 (17.44)** | **802 (15.80)** | **322.08 ± 80.92** | **245.00 ± 86.91** | **0.08** |
| Chloroflexi | 4 (0.05) | 0 | 4 (0.08) | 0 | 0.83 ± 0.72 | 0.03 |
| Cyanobacteria | 106 (1.34) | 27 (0.49) | 98 (1.93) | 7.17 ± 1.95 | 34.67 ± 7.85 | 0.00 |
| Firmicutes | 87 (1.10) | 68 (1.22) | 28 (0.55) | 15.83 ± 14.47 | 4.92 ± 3.45 | 0.01 |
| Fusobacteria | 14 (0.18) | 13 (0.23) | 1 (0.02) | 2.50 ± 2.71 | 0.17 ± 0.38 | 0.01 |
| Gemmatimonadetes | 16 (0.20) | 6 (0.11) | 14 (0.28) | 2.25 ± 2.45 | 3.08 ± 2.31 | 0.20 |
| Nitrospira | 21 (0.27) | 1 (0.02) | 21 (0.41) | 0.33 ± 0.49 | 4.83 ± 3.41 | 0.00 |
| OD1 | 5 (0.06) | 3 (0.05) | 2 (0.04) | 0.58 ± 1.08 | 0.50 ± 0.67 | 0.48 |
| Planctomycetes | 74 (0.94) | 50 (0.90) | 50 (0.98) | 17.25 ± 12.05 | 20.67 ± 9.21 | 0.02 |
| **Proteobacteria** | **3962 (50.13)** | **2821 (50.77)** | **2499 (49.22)** | **995.83 ± 188.88** | **822.42 ± 96.01** | **0.75** |
| Synergistetes | 4 (0.05) | 3 (0.05) | 1 (0.02) | 0.67 ± 0.98 | 0.17 ± 0.39 | 0.14 |
| TM7 | 2 (0.03) | 2 (0.04) | 0 | 0.33 ± 0.49 | 0 | 0.07 |
| unclassified | 721 (9.12) | 413 (7.43) | 523 (10.30) | 138.08 ± 62.53 | 175.17 ± 37.40 | 0.36 |
| **Verrucomicrobia** | **276 (3.49)** | **184 (3.31)** | **195 (3.84)** | **67.92 ± 31.40** | **75.83 ± 12.33** | **0.01** |
| WS3 | 5 (0.06) | 4 (0.07) | 5 (0.10) | 0.67 ± 0.89 | 2.25 ± 1.48 | 0.04 |
| Total | 7904 (100) | 5557 (100) | 5077 (100) | 2018.75 ± 404.75 | 1747.08 ± 115.19 | 0.00 |

^a^ Data represent total numbers of OTUs detected by pyrosequencing across all 24 samples.

^b^ Data represent total numbers of OTUs detected using 12 samples under dry or wet seasons.

^c^ Data represent average numbers of OTUs detected using 12 samples under dry or wet seasons.

^d^ unpaired t test between the dry and wet seasons using 12 samples under dry or wet seasons

^e^ The bold mean that higher number of detectable OTUs derived from some main phylum.
