# Supplementary material for: Seasonality Affects the Diversity and Composition of Bacterioplankton Communities in Dongjiang River, a Drinking Water Source of Hong Kong
Source: Front Microbiol. 2017 Aug 31;8:1644. doi: 10.3389/fmicb.2017.01644 (PMC5583224; doi:10.3389/fmicb.2017.01644)
Supplement: Supplementary file 6 [file Table6.DOCX]

**Table S6 Numbers of shared OTUs that were significantly (*p* <0.05) changed between the dry and wet season based on scaled abundances and unique OTUs detected only in dry or wet season.**

| Domain | Phylum | Shared OTUs | | Unique OTUs | |
| --- | --- | --- | --- | --- | --- |
|  |  | Down-wet^a^ | Up-wet^b^ | Dry season | Wet season |
| Bacteria | Acidobacteria | 0 | 6 | 5 | 25 |
|  | Actinobacteria | 120 | 110 | 419 | 240 |
|  | Armatimonadetes | 1 | 1 | 4 | 4 |
|  | Bacteroidetes | 77 | 62 | 543 | 376 |
|  | Chloroflexi | 0 | 0 | 0 | 4 |
|  | Cyanobacteria | 0 | 8 | 8 | 79 |
|  | Firmicutes | 3 | 0 | 59 | 19 |
|  | Fusobacteria | 0 | 0 | 13 | 1 |
|  | Gemmatimonadetes | 2 | 1 | 2 | 10 |
|  | Nitrospira | 1 | 0 | 0 | 20 |
|  | OD1 | 0 | 0 | 3 | 2 |
|  | Planctomycetes | 4 | 6 | 24 | 24 |
|  | Proteobacteria | 158 | 191 | 1463 | 1141 |
|  | Synergistetes | 0 | 0 | 3 | 1 |
|  | TM7 | 0 | 0 | 2 | 0 |
|  | unclassified | 23 | 40 | 198 | 308 |
|  | Verrucomicrobia | 6 | 14 | 81 | 92 |
|  | WS3 | 0 | 1 | 0 | 1 |
|  | Total | 395 | 440 | 2827 | 2347 |

a: Down-wet means the shared OTUs were significantly lower in wet season than in dry season samples by unpaired Student T tests.

b: Up-wet means the shared OTUs were significantly higher in wet season than in dry season samples by unpaired Student T tests.
