# Supplementary material for: Seasonality Affects the Diversity and Composition of Bacterioplankton Communities in Dongjiang River, a Drinking Water Source of Hong Kong
Source: Front Microbiol. 2017 Aug 31;8:1644. doi: 10.3389/fmicb.2017.01644 (PMC5583224; doi:10.3389/fmicb.2017.01644)
Supplement: Supplementary file 7 [file Table7.DOCX]

Table S7 Partial Mantel analyses of the relationship between the relative abundance of phylum and chemical or physical water properties ^a^.

|  | Chemical^b^ partial Physical^c^ properties | | Physical partial Chemical properties | |
| --- | --- | --- | --- | --- |
| Phylum | r | *P* | r | *P* |
| All detected OTUs | 0.540 | **0.001** | 0.234 | **0.022** |
| Acidobacteria | 0.450 | **0.001** | -0.081 | 0.708 |
| Actinobacteria | 0.521 | **0.001** | 0.483 | **0.001** |
| Armatimonadetes | 0.484 | **0.001** | -0.246 | 0.994 |
| Bacteroidetes | -0.037 | 0.585 | 0.365 | **0.008** |
| Nitrospira | 0.488 | **0.001** | -0.176 | 0.930 |
| Proteobacteria | 0.518 | **0.001** | -0.042 | 0.597 |
| Planctomycetes | 0.332 | **0.002** | 0.232 | **0.035** |
| WS3 | 0.426 | **0.002** | -0.124 | 0.850 |
| Cyanobacteria | 0.352 | **0.006** | 0.103 | 0.167 |
| Gemmatimonadetes | 0.329 | **0.007** | 0.173 | 0.101 |
| Firmicutes | 0.259 | **0.035** | -0.032 | 0.548 |
| Verrucomicrobia | -0.200 | 0.956 | 0.527 | **0.001** |

^a^ Only significantly (P<0.05) changed phylotype are shown in bold font.

^b^ Selected chemical properties included the concentrations of NH_4_^+^, NO_3_^-^, and TOC.

^c^ Selected physical properties included the TSS, pH, and temperature.
