# Supplementary material for: Seasonality Affects the Diversity and Composition of Bacterioplankton Communities in Dongjiang River, a Drinking Water Source of Hong Kong
Source: Front Microbiol. 2017 Aug 31;8:1644. doi: 10.3389/fmicb.2017.01644 (PMC5583224; doi:10.3389/fmicb.2017.01644)
Supplement: Supplementary file 8 [file Table8.DOCX]

Table S8 Partial Mantel analyses of the relationship between the relative abundance of class and chemical or physical water properties ^a^.

|  |  | Chemical^b^ partial Physical^c^ properties | | Physical partial Chemical properties | |
| --- | --- | --- | --- | --- | --- |
| Phylum | Class | r | *P* | r | *P* |
| Actinobacteria | Actinobacteria | 0.521 | **0.001** | 0.483 | **0.001** |
| Acidobacteria | unclassified | 0.420 | **0.001** | -0.142 | 0.933 |
|  | Holophagae | 0.507 | **0.001** | -0.159 | 0.924 |
|  | Acidobacteria_Gp3 | 0.282 | **0.013** | 0.026 | 0.351 |
| Armatimonadetes | Armatimonadia | 0.480 | **0.001** | -0.242 | 0.998 |
| Bacteroidetes | Bacteroidetes_incertae_sedis | 0.313 | **0.012** | -0.031 | 0.498 |
|  | unclassified | -0.020 | 0.538 | 0.579 | **0.001** |
|  | Sphingobacteria | 0.145 | 0.076 | 0.214 | **0.023** |
| Cyanobacteria | Cyanobacteria | 0.351 | **0.006** | 0.104 | 0.176 |
| Firmicutes | Bacilli | 0.277 | **0.025** | -0.006 | 0.494 |
| Gemmatimonadetes | Gemmatimonadetes | 0.329 | **0.010** | 0.173 | 0.106 |
| Nitrospira | Nitrospira | 0.488 | **0.001** | -0.176 | 0.935 |
| Planctomycetes | Planctomycetacia | 0.333 | **0.005** | 0.210 | 0.057 |
| Proteobacteria | Alphaproteobacteria | 0.491 | **0.001** | 0.160 | 0.053 |
|  | Betaproteobacteria | 0.510 | **0.001** | -0.045 | 0.575 |
|  | Gammaproteobacteria | 0.670 | **0.001** | 0.094 | 0.203 |
|  | unclassified | 0.428 | **0.002** | 0.002 | 0.438 |
|  | Deltaproteobacteria | 0.358 | **0.003** | -0.115 | 0.809 |
| Synergistetes | Synergistia | 0.229 | **0.049** | -0.120 | 0.808 |
| Verrucomicrobia | Subdivision3 | -0.167 | 0.926 | 0.615 | **0.001** |
|  | Opitutae | -0.147 | 0.875 | 0.393 | **0.007** |
| WS3 | WS3_genera_incertae_sedis | 0.426 | **0.003** | -0.124 | 0.841 |

^a^ Only significantly (*P* < 0.05) changed phylotype are shown in bold font.

^b^ Selected chemical properties included the concentrations of NH_4_^+^, NO_3_^-^, and TOC.

^c^ Selected physical properties included the TSS, pH, and temperature.
