# Supplementary material for: Seasonality Affects the Diversity and Composition of Bacterioplankton Communities in Dongjiang River, a Drinking Water Source of Hong Kong
Source: Front Microbiol. 2017 Aug 31;8:1644. doi: 10.3389/fmicb.2017.01644 (PMC5583224; doi:10.3389/fmicb.2017.01644)
Supplement: Supplementary file 9 [file Table9.DOCX]

Table S9 Partial Mantel analyses of the relationship between the relative abundance of order and chemical or physical water properties ^a^.

|  |  |  | Chemical^b^ partial Physical^c^ properties | | Physical partial Chemical properties | |
| --- | --- | --- | --- | --- | --- | --- |
| Phylum | Class | Order | r | *P* ^d^ | r | *P* |
| Acidobacteria | Acidobacteria_Gp3 | Gp3 | 0.282 | **0.018** | 0.026 | 0.373 |
|  | Holophagae | Holophagales | 0.507 | **0.001** | -0.159 | 0.928 |
| Actinobacteria | Actinobacteria | unclassified | 0.514 | **0.001** | 0.089 | 0.191 |
|  |  | Actinomycetales | 0.420 | **0.001** | 0.480 | **0.001** |
|  |  | Coriobacteriales | 0.412 | **0.005** | -0.271 | 0.997 |
| Armatimonadetes | Armatimonadia | Armatimonadales | 0.480 | **0.001** | -0.242 | 0.996 |
| Bacteroidetes | Bacteroidetes_incertae_sedis | Ohtaekwangia | 0.313 | **0.010** | -0.031 | 0.518 |
|  | Sphingobacteria | Sphingobacteriales | 0.145 | 0.068 | 0.214 | **0.015** |
| Cyanobacteria | Cyanobacteria | Family_II | 0.358 | **0.004** | 0.109 | 0.167 |
| Firmicutes | Bacilli | unclassified | 0.443 | **0.003** | -0.236 | 0.992 |
|  |  | Lactobacillales | 0.352 | **0.006** | -0.070 | 0.670 |
| Gemmatimonadetes | Gemmatimonadetes | Gemmatimonadales | 0.329 | **0.007** | 0.173 | 0.122 |
| Nitrospira | Nitrospira | Nitrospirales | 0.488 | **0.001** | -0.176 | 0.947 |
| Planctomycetes | Planctomycetacia | Planctomycetales | 0.333 | **0.006** | 0.210 | **0.042** |
| Proteobacteria | Alphaproteobacteria | Rhizobiales | 0.593 | **0.001** | 0.050 | 0.315 |
|  |  | unclassified | 0.371 | **0.007** | -0.054 | 0.620 |
|  |  | Caulobacterales | 0.368 | **0.010** | -0.162 | 0.889 |
|  |  | Rhodospirillales | 0.255 | **0.039** | -0.021 | 0.509 |
|  |  | Alphaproteobacteria_incertae_sedis | -0.117 | 0.773 | 0.453 | **0.003** |
|  | Betaproteobacteria | unclassified | 0.496 | **0.001** | -0.220 | 0.975 |
|  |  | Rhodocyclales | 0.455 | **0.002** | -0.192 | 0.954 |
|  |  | Methylophilales | 0.315 | **0.005** | 0.053 | 0.277 |
|  |  | Nitrosomonadales | 0.332 | **0.012** | -0.147 | 0.872 |
|  |  | Burkholderiales | 0.118 | 0.149 | 0.298 | **0.016** |
|  |  | Hydrogenophilales | 0.041 | 0.358 | 0.291 | **0.031** |
|  | Deltaproteobacteria | unclassified | 0.389 | **0.005** | -0.118 | 0.819 |
|  |  | Myxococcales | 0.268 | **0.024** | 0.014 | 0.428 |
|  |  | Bdellovibrionales | 0.280 | **0.027** | -0.214 | 0.970 |
|  | Gammaproteobacteria | Enterobacteriales | 0.468 | **0.002** | -0.237 | 0.986 |
|  |  | Xanthomonadales | 0.341 | **0.003** | -0.193 | 0.978 |
|  |  | Methylococcales | 0.340 | **0.007** | -0.097 | 0.749 |
|  |  | Legionellales | 0.386 | **0.011** | -0.043 | 0.566 |
|  |  | Chromatiales | -0.317 | 1.000 | 0.585 | **0.001** |
|  |  | unclassified | 0.594 | **0.001** | 0.205 | **0.046** |
| TM7 | TM7_genera_incertae_sedis | unclassified | -0.123 | 0.807 | 0.430 | **0.015** |
| Verrucomicrobia | Opitutae | Opitutales | -0.164 | 0.917 | 0.494 | **0.002** |
|  | Subdivision3 | Subdivision3_genera_incertae_sedis | -0.167 | 0.922 | 0.615 | **0.001** |

^a^ Only significantly (*P* < 0.05) changed phylotype are shown in bold font.

^b^ Selected chemical properties included the concentrations of NH_4_^+^, NO_3_^-^, and TOC.

^c^ Selected physical properties included the TSS, pH, and temperature.
