# Supplementary material for: Seasonality Affects the Diversity and Composition of Bacterioplankton Communities in Dongjiang River, a Drinking Water Source of Hong Kong
Source: Front Microbiol. 2017 Aug 31;8:1644. doi: 10.3389/fmicb.2017.01644 (PMC5583224; doi:10.3389/fmicb.2017.01644)
Supplement: Supplementary file 10 [file Table10.DOCX]

Table S10 Partial Mantel analyses of the relationship between the relative abundance of family and chemical or physical water properties ^a^.

|  |  |  |  | Chemical^b^ partial Physical^c^ properties | | Physical partial Chemical properties | |
| --- | --- | --- | --- | --- | --- | --- | --- |
| Phylum | Class | Order | Family | r | *P* | r | *P* |
| Acidobacteria | Acidobacteria_Gp3 | Gp3 | unclassified | 0.282 | **0.021** | 0.026 | 0.391 |
|  | Holophagae | Holophagales | Holophagaceae | 0.507 | **0.001** | -0.159 | 0.911 |
|  | Acidobacteria_Gp16 | Gp16 | unclassified | -0.100 | 0.758 | 0.229 | **0.044** |
| Actinobacteria | Actinobacteria | Actinomycetales | Actinomycetaceae | 0.476 | **0.003** | -0.255 | 0.990 |
|  |  |  | unclassified | 0.422 | **0.001** | 0.462 | **0.001** |
|  |  |  | Nocardiaceae | -0.161 | 0.868 | 0.509 | **0.003** |
|  |  | Acidimicrobiales | unclassified | 0.144 | 0.055 | 0.385 | **0.001** |
|  |  | Coriobacteriales | Coriobacteriaceae | 0.412 | **0.003** | -0.271 | 0.995 |
|  |  | Solirubrobacterales | Conexibacteraceae | 0.222 | **0.050** | 0.062 | 0.254 |
| Armatimonadetes | Armatimonadia | Armatimonadales | Armatimonadaceae | 0.480 | **0.001** | -0.242 | 0.998 |
| Bacteroidetes | Bacteroidia | Bacteroidales | Prevotellaceae | 0.435 | **0.001** | -0.237 | 0.981 |
|  |  |  | Rikenellaceae | 0.498 | **0.001** | -0.247 | 0.995 |
|  | Sphingobacteria | Sphingobacteriales | unclassified | 0.402 | **0.001** | -0.071 | 0.714 |
|  |  |  | Sphingobacteriaceae | 0.318 | **0.004** | 0.097 | 0.220 |
|  |  |  | Saprospiraceae | -0.258 | 0.992 | 0.452 | **0.002** |
|  |  |  | Cytophagaceae | 0.046 | 0.265 | 0.219 | **0.035** |
| Cyanobacteria_Chloroplast | Cyanobacteria | Family_II | GpIIa | 0.358 | **0.002** | 0.109 | 0.162 |
| Firmicutes | Bacilli | Lactobacillales | Streptococcaceae | 0.476 | **0.001** | -0.214 | 0.977 |
|  | Clostridia | Clostridiales | Lachnospiraceae | 0.327 | **0.011** | -0.224 | 0.968 |
| Gemmatimonadetes | Gemmatimonadetes | Gemmatimonadales | Gemmatimonadaceae | 0.329 | **0.009** | 0.173 | 0.092 |
| Nitrospira | Nitrospira | Nitrospirales | Nitrospiraceae | 0.488 | **0.001** | -0.176 | 0.943 |
| Planctomycetes | Planctomycetacia | Planctomycetales | Planctomycetaceae | 0.334 | **0.003** | 0.210 | 0.057 |
| Proteobacteria | Alphaproteobacteria | Caulobacterales | Caulobacteraceae | 0.368 | **0.008** | -0.162 | 0.890 |
|  |  | Rhizobiales | Hyphomicrobiaceae | 0.562 | **0.001** | -0.367 | 1.000 |
|  |  |  | unclassified | 0.421 | **0.001** | 0.237 | **0.025** |
|  |  |  | Methylocystaceae | 0.468 | **0.002** | 0.324 | **0.022** |
|  |  |  | Rhizobiales_incertae_sedis | 0.364 | **0.006** | -0.182 | 0.943 |
|  |  | Alphaproteobacteria_incertae_sedis | Rhizomicrobium | -0.117 | 0.794 | 0.453 | **0.006** |
|  |  | Rhodospirillales | Acetobacteraceae | 0.244 | **0.036** | -0.023 | 0.467 |
|  | Betaproteobacteria | Burkholderiales | Burkholderiaceae | 0.374 | **0.003** | 0.208 | **0.047** |
|  |  |  | Sutterellaceae | 0.379 | **0.004** | -0.172 | 0.946 |
|  |  |  | Alcaligenaceae | -0.080 | 0.711 | 0.620 | **0.001** |
|  |  |  | Comamonadaceae | 0.054 | 0.297 | 0.280 | **0.027** |
|  |  | Hydrogenophilales | Hydrogenophilaceae | 0.041 | 0.342 | 0.291 | **0.031** |
|  |  | Methylophilales | Methylophilaceae | 0.315 | **0.004** | 0.053 | 0.296 |
|  |  | Nitrosomonadales | Nitrosomonadaceae | 0.330 | **0.017** | -0.146 | 0.893 |
|  |  | Rhodocyclales | Rhodocyclaceae | 0.455 | **0.002** | -0.192 | 0.955 |
|  | Deltaproteobacteria | Bdellovibrionales | Bacteriovoracaceae | 0.353 | **0.002** | -0.227 | 0.997 |
|  |  | Desulfuromonadales | Desulfuromonadaceae | 0.322 | **0.031** | -0.173 | 0.934 |
|  |  | Myxococcales | Polyangiaceae | 0.268 | **0.026** | -0.023 | 0.530 |
|  |  |  | unclassified | 0.259 | **0.040** | 0.046 | 0.347 |
|  | Gammaproteobacteria | Enterobacteriales | Enterobacteriaceae | 0.468 | **0.001** | -0.237 | 0.992 |
|  |  | Legionellales | Legionellaceae | 0.386 | **0.007** | -0.043 | 0.553 |
|  |  | Methylococcales | Methylococcaceae | 0.340 | **0.009** | -0.097 | 0.752 |
|  |  | Pseudomonadales | Moraxellaceae | 0.277 | **0.026** | -0.134 | 0.835 |
|  |  | Xanthomonadales | Sinobacteraceae | 0.381 | **0.005** | -0.168 | 0.914 |
|  |  | Chromatiales | Chromatiaceae | -0.317 | 0.999 | 0.585 | **0.002** |
| Verrucomicrobia | Opitutae | Opitutales | Opitutaceae | -0.164 | 0.921 | 0.494 | **0.002** |
|  | Subdivision3 | Subdivision3_genera_incertae_sedis | unclassified | -0.167 | 0.926 | 0.615 | **0.001** |

^a^ Only significantly (*P* < 0.05) changed phylotype are shown in bold font.

^b^ Selected chemical properties included the concentrations of NH_4_^+^, NO_3_^-^, and TOC.

^c^ Selected physical properties included the TSS, pH, and temperature.
