# Supplementary material for: Seasonality Affects the Diversity and Composition of Bacterioplankton Communities in Dongjiang River, a Drinking Water Source of Hong Kong
Source: Front Microbiol. 2017 Aug 31;8:1644. doi: 10.3389/fmicb.2017.01644 (PMC5583224; doi:10.3389/fmicb.2017.01644)
Supplement: Supplementary file 11 [file Table11.DOCX]

Table S11 Partial Mantel analyses of the relationship between the relative abundance of genus and chemical or physical water properties ^a^.

|  |  |  |  |  | Chemical^b^ partial Physical^c^ properties | | Physical partial Chemical properties | |
| --- | --- | --- | --- | --- | --- | --- | --- | --- |
| Phylum | Class | Order | Family | Genus | r | *P* | r | *P* |
| Acidobacteria | Holophagae | Holophagales | Holophagaceae | unclassified | 0.507 | **0.002** | -0.159 | 0.939 |
| Actinobacteria | Actinobacteria | Actinomycetales | Actinomycetaceae | Actinomyces | 0.476 | **0.001** | -0.255 | 0.990 |
|  |  |  | Microbacteriaceae | Leucobacter | 0.371 | **0.019** | -0.174 | 0.942 |
|  |  |  |  | unclassified | 0.377 | **0.002** | 0.202 | 0.076 |
|  |  |  |  | Microbacterium | -0.203 | 0.949 | 0.350 | **0.021** |
|  |  |  | Acidimicrobiaceae | unclassified | 0.010 | 0.432 | 0.381 | **0.003** |
|  |  |  | Nocardiaceae | Gordonia | -0.161 | 0.869 | 0.509 | **0.002** |
|  |  | Coriobacteriales | Coriobacteriaceae | Atopobium | 0.351 | **0.012** | -0.138 | 0.882 |
|  |  |  |  | Collinsella | 0.352 | **0.006** | -0.258 | 1.000 |
| Armatimonadetes | Armatimonadia | Armatimonadales | Armatimonadaceae | Armatimonas_Armatimonadetes_gp1 | 0.480 | **0.001** | -0.242 | 0.996 |
| Bacteroidetes | Bacteroidia | Bacteroidales | Prevotellaceae | Prevotella | 0.435 | **0.001** | -0.241 | 0.988 |
|  |  |  | Rikenellaceae | Alistipes | 0.498 | **0.003** | -0.247 | 0.995 |
|  | Sphingobacteria | Sphingobacteriales | Chitinophagaceae | Sediminibacterium | 0.342 | **0.008** | 0.030 | 0.366 |
|  |  |  | Cytophagaceae | Emticicia | 0.344 | **0.009** | -0.214 | 0.974 |
|  |  |  |  | unclassified | 0.050 | 0.268 | 0.223 | **0.022** |
|  |  |  | Saprospiraceae | Haliscomenobacter | -0.145 | 0.902 | 0.260 | **0.028** |
|  |  |  |  | unclassified | -0.223 | 0.968 | 0.421 | **0.004** |
|  |  |  | Sphingobacteriaceae | Sphingobacterium | 0.322 | **0.029** | -0.173 | 0.951 |
| Cyanobacteria | Cyanobacteria | Family_II | GpIIa | unclassified | 0.358 | **0.001** | 0.109 | 0.164 |
| Firmicutes | Bacilli | Lactobacillales | Carnobacteriaceae | Granulicatella | 0.430 | **0.001** | -0.234 | 0.996 |
|  |  |  | Streptococcaceae | Streptococcus | 0.486 | **0.001** | -0.238 | 0.99 |
|  | Clostridia | Clostridiales | Lachnospiraceae | Clostridium_XlVa | 0.379 | **0.009** | -0.172 | 0.954 |
|  |  |  |  | Lachnospiracea_incertae_sedis | 0.366 | **0.007** | -0.240 | 0.993 |
|  |  |  |  | Oribacterium | 0.397 | **0.004** | -0.252 | 0.999 |
|  |  |  |  | Roseburia | 0.371 | **0.011** | -0.174 | 0.954 |
|  |  |  |  | unclassified | 0.293 | **0.028** | -0.109 | 0.84 |
|  |  |  | Ruminococcaceae | Faecalibacterium | 0.278 | **0.027** | -0.189 | 0.938 |
|  |  |  | Veillonellaceae | Megasphaera | 0.356 | **0.011** | -0.199 | 0.974 |
|  |  |  |  | Veillonella | 0.512 | **0.001** | -0.258 | 0.996 |
| Fusobacteria | Fusobacteria | Fusobacteriales | Fusobacteriaceae | Fusobacterium | 0.514 | **0.002** | -0.242 | 0.992 |
|  |  |  | Leptotrichiaceae | unclassified | 0.371 | **0.011** | -0.174 | 0.949 |
| Gemmatimonadetes | Gemmatimonadetes | Gemmatimonadales | Gemmatimonadaceae | Gemmatimonas | 0.329 | **0.008** | 0.173 | 0.104 |
| Nitrospira | Nitrospira | Nitrospirales | Nitrospiraceae | Nitrospira | 0.488 | **0.001** | -0.176 | 0.937 |
| Planctomycetes | Planctomycetacia | Planctomycetales | Planctomycetaceae | Schlesneria | 0.320 | **0.012** | -0.067 | 0.663 |
|  |  |  |  | Singulisphaera | 0.371 | **0.023** | -0.174 | 0.940 |
|  |  |  |  | unclassified | 0.319 | **0.013** | 0.170 | 0.085 |
|  |  |  |  | Gemmata | 0.170 | 0.052 | 0.271 | **0.014** |
|  |  |  |  | Planctomyces | -0.362 | 1.000 | 0.606 | **0.001** |
| Proteobacteria | Alphaproteobacteria | Alphaproteobacteria_incertae_sedis | Rhizomicrobium | unclassified | -0.117 | 0.787 | 0.453 | **0.004** |
|  |  | Caulobacterales | Caulobacteraceae | Caulobacter | 0.275 | **0.035** | -0.177 | 0.921 |
|  |  |  |  | Phenylobacterium | 0.365 | **0.01** | -0.116 | 0.774 |
|  |  |  |  | unclassified | 0.371 | **0.014** | -0.174 | 0.954 |
|  |  | Rhizobiales | Hyphomicrobiaceae | Hyphomicrobium | 0.530 | **0.001** | -0.344 | 1.000 |
|  |  |  |  | unclassified | 0.451 | **0.002** | -0.266 | 0.999 |
|  |  |  | Bradyrhizobiaceae | Bosea | -0.002 | 0.481 | 0.369 | **0.011** |
|  |  |  | Methylobacteriaceae | Methylobacterium | -0.244 | 0.989 | 0.324 | **0.022** |
|  |  |  | Methylocystaceae | Methylocystis | 0.468 | **0.001** | -0.158 | 0.918 |
|  |  |  | Rhizobiales_incertae_sedis | Vasilyevaea | 0.364 | **0.011** | -0.182 | 0.951 |
|  |  | Rhodobacterales | Rhodobacteraceae | Rhodobacter | -0.113 | 0.804 | 0.252 | **0.041** |
|  |  | Rhodospirillales | Acetobacteraceae | Acetobacter | 0.379 | **0.008** | -0.172 | 0.951 |
|  |  |  |  | unclassified | 0.242 | **0.049** | -0.010 | 0.504 |
|  |  | Sphingomonadales | Sphingomonadaceae | Sphingobium | 0.359 | **0.012** | -0.190 | 0.966 |
|  | Betaproteobacteria | Burkholderiales | Burkholderiaceae | Polynucleobacter | 0.373 | **0.002** | 0.209 | **0.042** |
|  |  |  | Alcaligenaceae | Derxia | -0.235 | 0.99 | 0.620 | **0.002** |
|  |  |  | Alcaligenaceae | Pigmentiphaga | -0.284 | 0.999 | 0.557 | **0.001** |
|  |  |  | Comamonadaceae | Albidiferax | 0.046 | 0.328 | 0.526 | **0.001** |
|  |  |  |  | Kinneretia | -0.29 | 0.998 | 0.525 | **0.004** |
|  |  |  |  | Limnohabitans | 0.029 | 0.377 | 0.308 | **0.024** |
|  |  |  |  | Polaromonas | -0.251 | 0.998 | 0.479 | **0.001** |
|  |  |  |  | Acidovorax | 0.225 | **0.043** | -0.011 | 0.499 |
|  |  |  |  | Diaphorobacter | 0.541 | **0.001** | -0.269 | 0.999 |
|  |  |  |  | Simplicispira | 0.315 | **0.011** | -0.185 | 0.941 |
|  |  | Hydrogenophilales | Hydrogenophilaceae | Thiobacillus | 0.0414 | 0.381 | 0.291 | **0.026** |
|  |  |  | Sutterellaceae | Parasutterella | 0.379 | **0.006** | -0.172 | 0.951 |
|  |  | Methylophilales | Methylophilaceae | Methylotenera | 0.319 | **0.004** | 0.042 | 0.333 |
|  |  | Neisseriales | Neisseriaceae | Formivibrio | 0.371 | **0.013** | -0.174 | 0.962 |
|  |  |  |  | Neisseria | 0.365 | **0.004** | -0.120 | 0.84 |
|  |  |  |  | Chromobacterium | -0.319 | 1 | 0.589 | **0.001** |
|  |  | Nitrosomonadales | Nitrosomonadaceae | unclassified | 0.337 | **0.007** | -0.149 | 0.884 |
|  |  | Rhodocyclales | Rhodocyclaceae | Methyloversatilis | 0.458 | **0.003** | -0.268 | 1.000 |
|  |  |  |  | Azospira | -0.270 | 0.995 | 0.516 | **0.006** |
|  |  |  |  | Sulfuritalea | -0.232 | 0.979 | 0.574 | **0.002** |
|  |  |  |  | unclassified | 0.457 | **0.001** | -0.197 | 0.977 |
|  | Deltaproteobacteria | Bdellovibrionales | Bacteriovoracaceae | Peredibacter | 0.353 | **0.003** | -0.227 | 0.996 |
|  |  | Desulfuromonadales | Desulfuromonadaceae | Desulfuromonas | 0.322 | **0.037** | -0.173 | 0.953 |
|  |  | Myxococcales | Polyangiaceae | unclassified | 0.303 | **0.018** | -0.083 | 0.708 |
|  | Gammaproteobacteria | Enterobacteriales | Enterobacteriaceae | unclassified | 0.468 | **0.001** | -0.237 | 0.993 |
|  |  | Chromatiales | Chromatiaceae | Rheinheimera | -0.317 | 1.000 | 0.585 | **0.001** |
|  |  | Legionellales | Legionellaceae | Legionella | 0.386 | **0.003** | -0.043 | 0.535 |
|  |  | Methylococcales | Methylococcaceae | Methylocaldum | 0.268 | **0.037** | -0.121 | 0.822 |
|  |  |  |  | Methylomonas | 0.474 | **0.001** | -0.274 | 0.998 |
|  |  |  |  | Methylosarcina | 0.356 | **0.017** | -0.199 | 0.977 |
|  |  |  |  | unclassified | 0.407 | **0.001** | -0.074 | 0.677 |
|  |  | Pseudomonadales | Moraxellaceae | Acinetobacter | 0.244 | **0.046** | -0.110 | 0.782 |
|  |  |  |  | unclassified | 0.281 | **0.034** | -0.105 | 0.811 |
|  |  | Xanthomonadales | Sinobacteraceae | Steroidobacter | 0.393 | **0.003** | -0.193 | 0.963 |
|  |  |  | Xanthomonadaceae | Thermomonas | 0.470 | **0.001** | -0.213 | 0.976 |
| Synergistetes | Synergistia | Synergistales | Synergistaceae | Cloacibacillus | 0.371 | **0.014** | -0.154 | 0.903 |
| Verrucomicrobia | Verrucomicrobiae | Verrucomicrobiales | Verrucomicrobiaceae | Luteolibacter | 0.494 | **0.001** | -0.266 | 0.995 |
|  |  |  |  | unclassified | 0.361 | **0.006** | -0.175 | 0.944 |
|  |  |  |  | Verrucomicrobium | 0.352 | **0.015** | -0.140 | 0.888 |
|  | Opitutae | Opitutales | Opitutaceae | Opitutus | -0.164 | 0.913 | 0.494 | **0.001** |

^a^ Only significantly (*P* < 0.05) changed phylotype are shown in bold font

^b^ Selected chemical properties included the concentrations of NH_4_^+^, NO_3_^-^, and TOC.

^c^ Selected physical properties included the TSS, pH, and temperature.
