# Supplementary material for: Seasonality Affects the Diversity and Composition of Bacterioplankton Communities in Dongjiang River, a Drinking Water Source of Hong Kong
Source: Front Microbiol. 2017 Aug 31;8:1644. doi: 10.3389/fmicb.2017.01644 (PMC5583224; doi:10.3389/fmicb.2017.01644)
Supplement: Supplementary file 13 [file Image2.PDF]

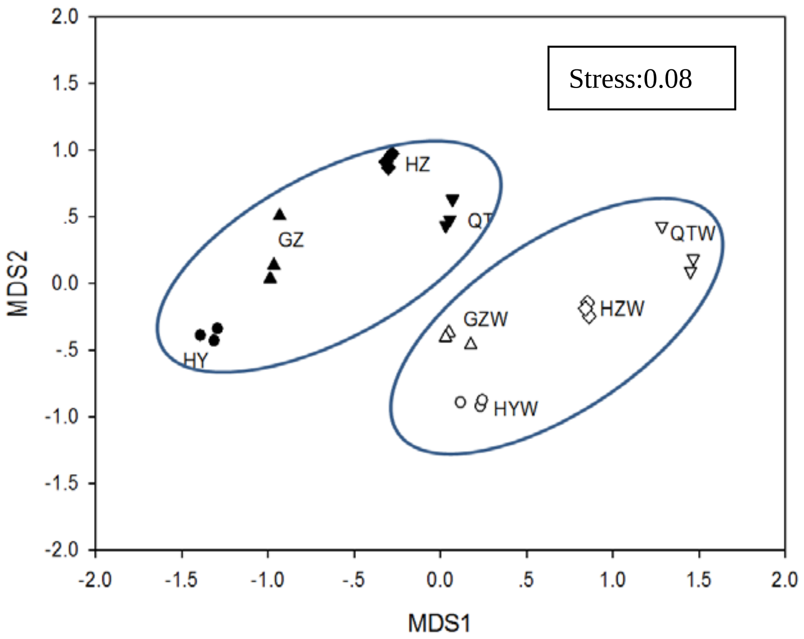

Figure S2 NMDS (nonmetric multidimensional scaling) plot of bacterial communities from the water samples of the Dongjiang River in dry and wet seasons. Points that are close together represent the water samples with similar bacterial community composition based on the pyrosequencing method. The solid and hollow symbols respectively indicated the water samples of the dry and wet seasons and each symbol shape represented samples from the same sampling sites. The solid ovals showed the two clusters of bacterial communities. The stress value of this ordination was 0.08, which indicated that the overall structure of the data set presented a good ordination [1]. No scales are shown on the axes because there are no meaningful absolute unites for the axes of MDS plots [1]; the relative distances between points represent the rank order of dissimilarities between samples[2].

Reference

1. Clarke KR, Warwick RM: **Change in Marine Communities: An Approach to Statistical Analysis and Interpretation, Second edition.**: PRIMER-E Limited, Plymouth,UK; 2001.

2. Fierer N, Morse JL, Berthrong ST, Bernhardt ES, Jackson RB: **Environmental controls on the landscape-scale biogeography of stream bacterial communities**. *Ecology* 2007, **88**(9):2162-2173.
