# Supplementary material for: Seasonality Affects the Diversity and Composition of Bacterioplankton Communities in Dongjiang River, a Drinking Water Source of Hong Kong
Source: Front Microbiol. 2017 Aug 31;8:1644. doi: 10.3389/fmicb.2017.01644 (PMC5583224; doi:10.3389/fmicb.2017.01644)
Supplement: Supplementary file 14 [file Image3.PDF]

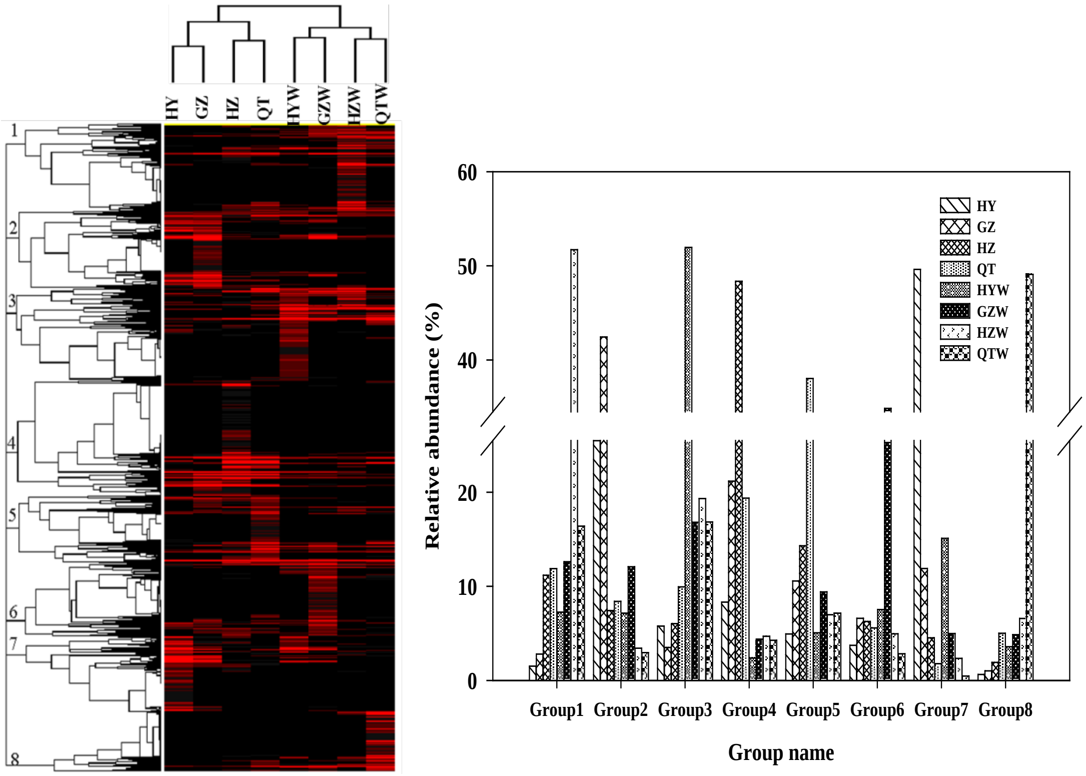

a b

Figure S3 Hierarchical clustering analysis of microbiological communities from the water samples of the Dongjiang River in dry and wet seasons based on the relative abundance of OTUs detected by barcoded pyrosequencing. The OTUs were defined with 3% dissimilarity. The figure was generated using Cluster and visualized in Treeview. The color scale indicates relative abundances. Eight samples were clearly separated into two groups and eight OTU groups were observed and indicated by numbers in the tree (a), and also illustrated in the graphs (b). The abbreviations represent the sampling sites Heyuan, Guzhu, Huizhou, and Qiaotou in dry (HY, GZ, HZ and QT) and wet seasons (HYW, GZW, HZW and QTW).
