# Supplementary material for: Seasonality Affects the Diversity and Composition of Bacterioplankton Communities in Dongjiang River, a Drinking Water Source of Hong Kong
Source: Front Microbiol. 2017 Aug 31;8:1644. doi: 10.3389/fmicb.2017.01644 (PMC5583224; doi:10.3389/fmicb.2017.01644)
Supplement: Supplementary file 15 [file Image4.PDF]

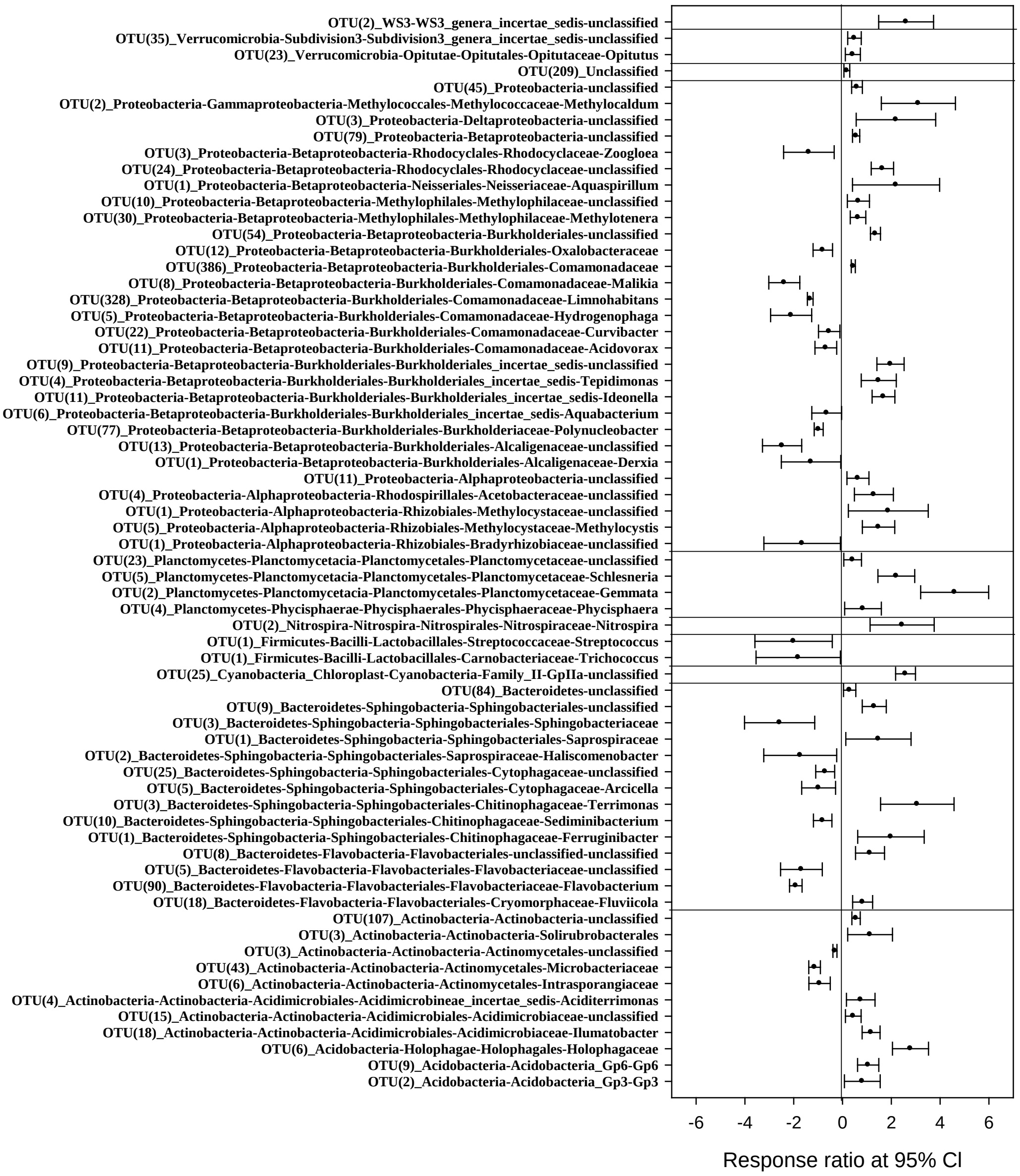

Figure S4 Significantly changed OTUs in the phyla of *Acidobacteria, Bacteroidetes, Cyanobacteria, Firmicutes, Nitrospira, Planctomycetes, Proteobacteria, Verrucomicrobia, WS3-WS3_genera* and unclassified phylotypes at wet season compared with dry season using the response ration method at 95% confidence interval.
