# Supplementary material for: Seasonality Affects the Diversity and Composition of Bacterioplankton Communities in Dongjiang River, a Drinking Water Source of Hong Kong
Source: Front Microbiol. 2017 Aug 31;8:1644. doi: 10.3389/fmicb.2017.01644 (PMC5583224; doi:10.3389/fmicb.2017.01644)
Supplement: Supplementary file 16 [file Image5.PDF]

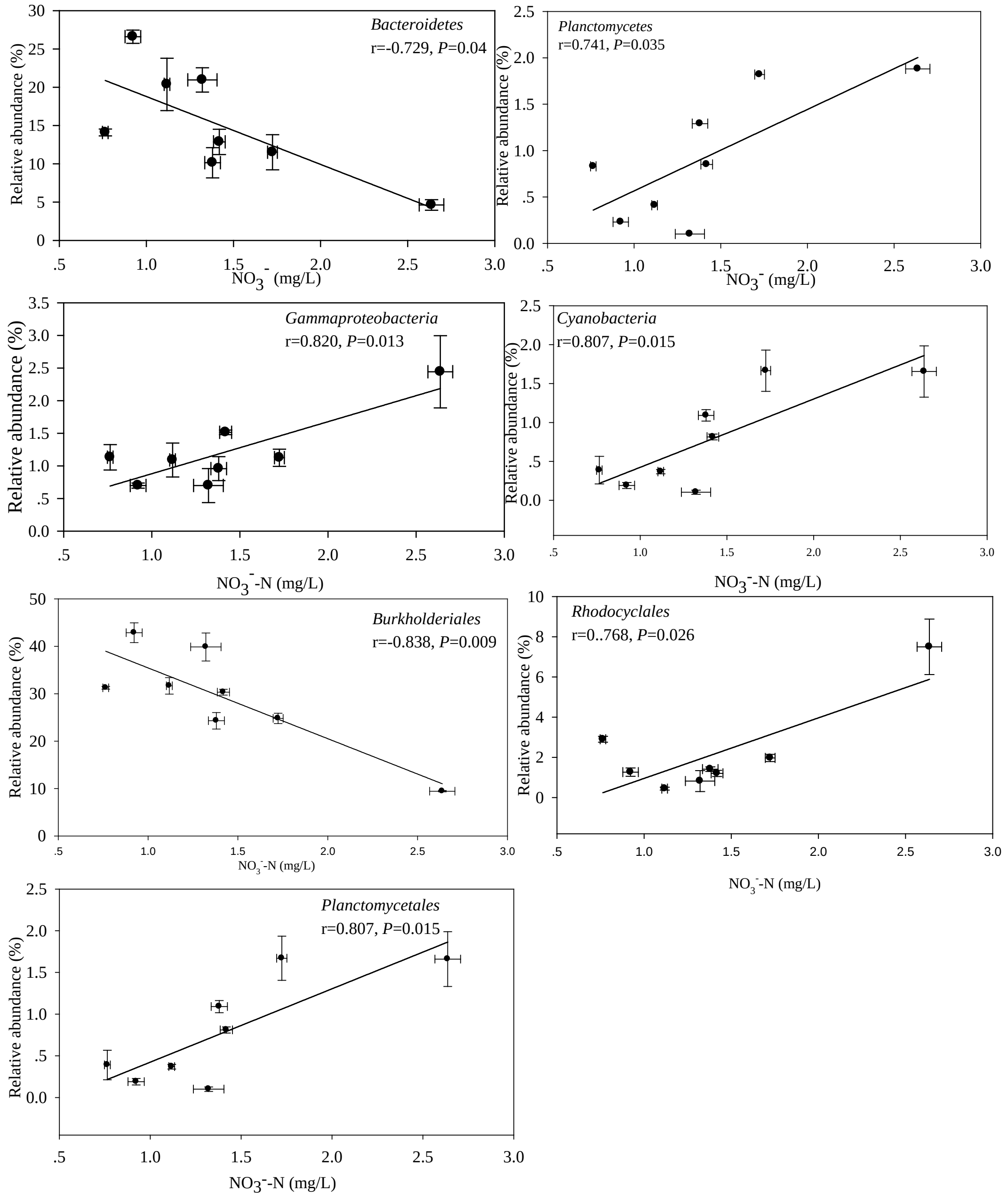

Figure S5 Relationships between relative abundances of dominant bacterial groups and the content of NO_3^--N. Linear regressions were used to test Pearson correlation between each taxon's relative abundance and the content of NO_3^--N at the levels of phylum, class and order.
