# Supplementary material for: Seasonality Affects the Diversity and Composition of Bacterioplankton Communities in Dongjiang River, a Drinking Water Source of Hong Kong
Source: Front Microbiol. 2017 Aug 31;8:1644. doi: 10.3389/fmicb.2017.01644 (PMC5583224; doi:10.3389/fmicb.2017.01644)
Supplement: Supplementary file 17 [file Image6.PDF]

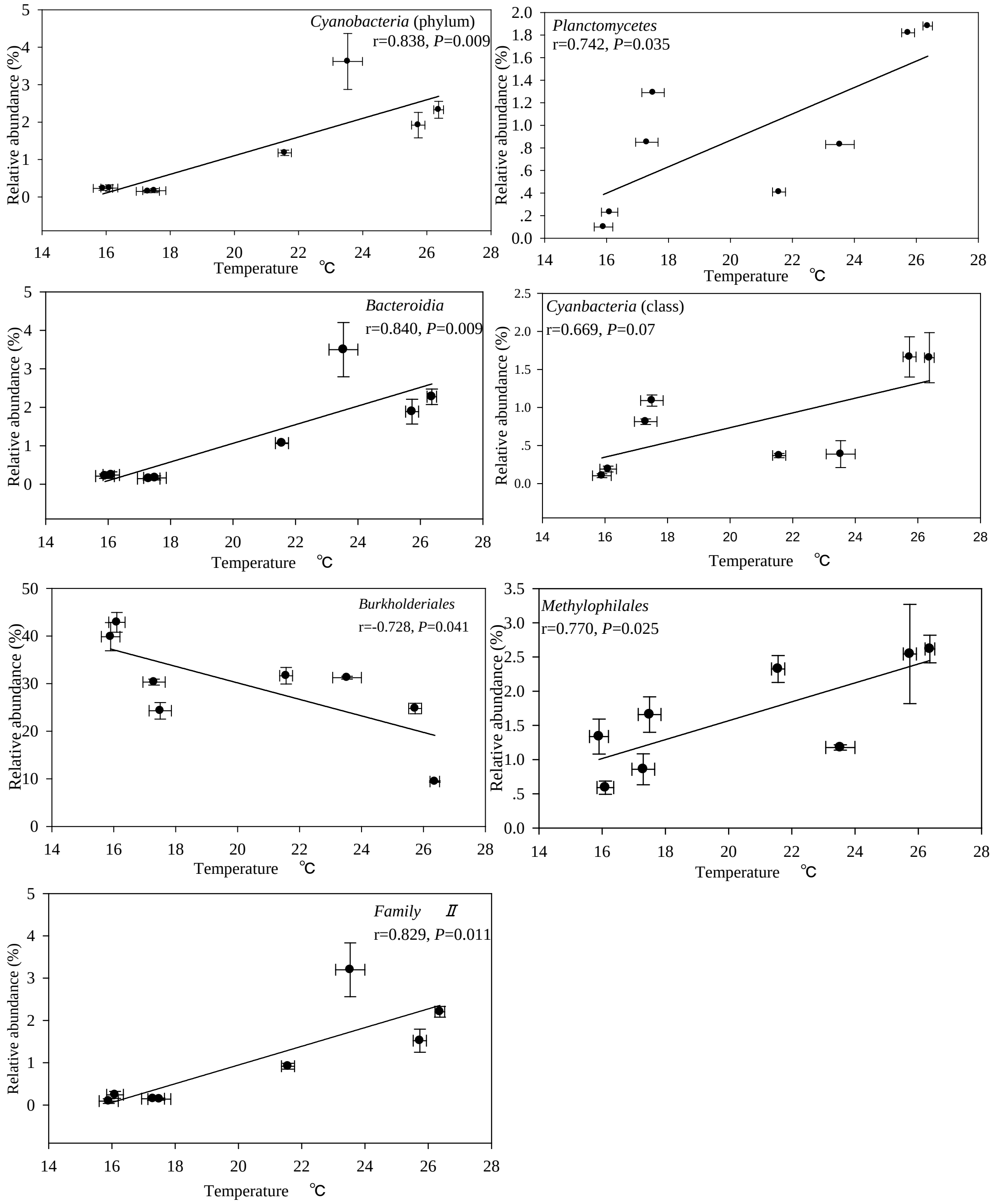

Figure S6 Relationships between relative abundances of dominant bacterial groups and temperature. Linear regressions were used to test Pearson correlation between each taxon's relative abundance and temperature at the levels of phylum, class and order.
